# Metabolic Profiling Reveals a Glycolytic Shift and an IRG1/Itaconate/NF2L2 Axis Regulating Neurotoxic Oxidative Stress in Inflammatory Microglia

**DOI:** 10.1111/jnc.70219

**Published:** 2025-09-05

**Authors:** Pinelopi Engskog‐Vlachos, Mikael K. R. Engskog, Martin Skandik, Kathleen Grabert, Noah Moruzzi, Marie‐Kim St‐Pierre, Ahmed M. Osman, Theodora Sylaidi, Klas Blomgren, Per‐Olof Berggren, Bertrand Joseph

**Affiliations:** ^1^ Toxicology Unit, Institute of Environmental Medicine Karolinska Institutet Stockholm Sweden; ^2^ Department of Medicinal Chemistry Uppsala University Uppsala Sweden; ^3^ The Rolf Luft Research Center for Diabetes and Endocrinology Karolinska Institutet Stockholm Sweden; ^4^ Department of Women's and Children's Health Karolinska Institutet Stockholm Sweden; ^5^ Center for Neuromusculoskeletal Restorative Medicine Shui On Centre Wan Chai Hong Kong

**Keywords:** central nervous system, immune activation, IRG1/ACOD1, itaconate, metabolic reprogramming, microglia, neuroinflammation, NF2L2/NRF2

## Abstract

Polar metabolic profiling, as well as bioenergetic assays, were used to characterize microglial responses to lipopolysaccharide, which induces a pro‐inflammatory state, and interleukin‐4, which is associated with an anti‐inflammatory phenotype. BV2 microglial cells and primary microglia were used for these investigations. Results revealed that lipopolysaccharide‐treated microglia exhibited an increased aerobic glycolytic activity measured by extracellular flux analysis, accompanied by increased levels of endogenous itaconate, a metabolite produced by the IRG1 enzyme. Increased itaconate levels observed by LC‐HRMS were found to be associated with a stabilization of the NF2L2/NRF2 transcription factor. Attenuation of the *Acod1* gene leads to increased pro‐inflammatory cytokine production, as measured by ELISA, while having no effect on LPS‐induced oxidative stress or neurotoxicity, an effect only observed upon silencing *Nfe2l2*. This suggests that an IRG1/itaconate/NRF2 axis functions as a feedback mechanism. The study underscores the dual role of metabolic reprogramming in microglial activation, balancing inflammation and neuroprotection, and suggests potential therapeutic targets for neuroinflammatory diseases by modulating itaconate and NF2L2/NRF2‐related pathways. This work highlights the complexity and therapeutic potential of targeting microglial metabolism in CNS disorders.

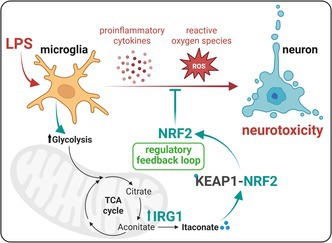

## Graphical abstract

Microglia, the brain's immune cells, change their metabolism depending on whether they are fighting infection or promoting healing. This study shows that during inflammation, microglia increase a molecule called itaconate, which helps reduce harmful stress in the brain while still allowing an immune response. These findings reveal a built‐in balancing system in microglia that could be targeted to treat neuroinflammatory diseases.

**Abbreviations**
ABCG2: ATP‐binding cassette sub‐family G member 2
ACOD1: aconitate decarboxylase 1
ACTB: beta‐actin
ARG1: arginase 1
ATF3: activating transcription factor 3
CCL2/MCP‐1: chemokine (C‐C motif) ligand 2/monocyte chemoattractant protein‐1
CCL3/MIP‐1α: chemokine (C‐C motif) ligand 3/macrophage inflammatory protein‐1 alpha
CCL4/MIP‐1β: chemokine (C‐C motif) ligand 4/macrophage inflammatory protein‐1 beta
CD11b: cluster of differentiation molecule 11b
CNS: central nervous system
CXCL10/IP‐10: chemokine (C‐X‐C motif) ligand 10/interferon gamma‐induced protein 10
CXCL9/MIG: chemokine (C‐X‐C motif) ligand 9/monokine induced by gamma interferon
ECAR: extracellular acidification rate
ESI: electrospray ionization
FA: formic acid
FBS: fetal bovine serum
FCCP: carbonyl cyanide‐4‐(trifluoromethoxy)phenylhydrazone
GM‐CSF: granulocyte‐macrophage colony‐stimulating factor
HMDB: human metabolome database
IFN‐α: interferon alpha
IFN‐γ: interferon gamma
IL10: interleukin 10
IL1β: interleukin 1 beta
IL4: interleukin 4
IL6: interleukin 6
IRG1: immune‐responsive gene 1
KEAP1: Kelch‐like ECH‐associated protein 1
LC‐HRMS: liquid chromatography–high resolution mass spectrometry
LLE: liquid–liquid extraction
LPS: lipopolysaccharide
M‐CSF: macrophage colony‐stimulating factor
MN9D: mouse neuroblastoma × rat embryonic midbrain dopaminergic neuron hybrid cell line
MSI: metabolomics standards initiative
Nfe2l2: nuclear factor, erythroid 2 like 2 gene
NLRP3: NACHT, LRR and PYD domains‐containing protein 3
NOS2/iNOS: nitric oxide synthase 2/inducible nitric oxide synthase
NRF2: nuclear factor erythroid 2–related factor 2
OCR: oxygen consumption rate
PCA: principal component analysis
PPP: pentose phosphate pathway
PQN: probabilistic quotient normalization
ROS: reactive oxygen species
SDH: succinate dehydrogenase
SMPDB: small molecule pathway database
TLR4: Toll‐Like Receptor 4
TNF‐α: tumor necrosis factor alpha
VEGF: vascular endothelial growth factor

## 1 Introduction

Microglia, resident myeloid cells of the central nervous system (CNS), play crucial roles during brain development and immune surveillance related to the maintenance of brain homeostasis or the response to challenges related to CNS injuries, infections, or disorders (Li and Barres 2018). Upon exposure to various stimuli, these specialized immune cells acquire distinctive reactive states associated with unique functions (Paolicelli et al. 2022). Microglia are sometimes described as the macrophages of the brain; however, these myeloid cells have a different origin, coming from yolk sac progenitors, as compared to the bone marrow‐derived macrophages found in peripheral tissues (Ginhoux et al. 2010). The different microglial reactive states are recognized to rely on different transcriptomes and proteomes, and thereby functions (Balak et al. 2024). Metabolic reprogramming, that is, the controlled cellular changes in metabolites concentrations, has emerged as one additional level of the control of acquisition of reactive states by immune cells, inclusive of peripheral macrophages (Chen et al. 2024; Tharp et al. 2024; Sun and Li 2017). However, the contributions of metabolic alterations to the activation of microglia toward different phenotypes remain poorly understood, mainly due to the lack of hypotheses that are generated based on analytically reliable data (Jha et al. 2019; Colonna and Butovsky 2017; Engskog et al. 2016). Nevertheless, metabolic reprogramming is increasingly recognized as a crucial aspect of microglial activation and function (Nair et al. 2019; Sabogal‐Guáqueta et al. 2023; Orihuela et al. 2016; Voloboueva et al. 2013). Dysregulated microglial metabolism has been implicated in neuroinflammatory conditions, neurodegenerative diseases, and neurodevelopmental disorders, highlighting the importance of elucidating the metabolic pathways underlying microglial responses (Strogulski et al. 2023; Loppi et al. 2023).

Previous studies have demonstrated roles for itaconate in regulating inflammatory responses in microglia and macrophages, including effects on glycolysis, mitochondrial stress, and neuroprotection in diverse model systems ranging from cell culture to animal injury paradigms (Voloboueva et al. 2013; Chausse et al. 2020; Kong et al. 2024; Liu et al. 2025). However, these investigations each had specific limitations. They did not directly address the very early activation phase of microglia in simple in vitro settings, nor did they systematically compare pro‐ versus anti‐inflammatory conditions or examine Nuclear Factor Erythroid 2‐Related Factor 2 (Nrf2) signaling as a downstream target of the endogenous Immune‐Responsive Gene 1 (IRG1)/itaconate pathway. Thus, the link between microglial itaconate production and Nrf2‐mediated antioxidant regulation remains incompletely defined.

To address these knowledge gaps, we employed metabolic profiling of the polar metabolome, combined with cellular assays, to investigate in detail the reprogramming of microglia in response to two classical immune stimuli: lipopolysaccharide (LPS), a Toll‐like receptor (TLR4) agonist, and interleukin‐4 (IL4). Whereas LPS treatment is reported to induce a pro‐inflammatory response in microglia, IL4 treatment is associated with an anti‐inflammatory phenotype (Zuiderwijk‐Sick et al. 2021; Burguillos et al. 2011). Liquid chromatography high‐resolution mass spectrometry (LC‐HRMS) analysis was performed in both positive and negative ionization modes to capture distinctive changes in the metabolome for the LPS‐ and IL4‐stimulated microglia. This profiling analysis, combined with real‐time extracellular flux analysis, revealed that increased glycolysis was associated with the pro‐inflammatory microglial activation states. A significant increase in the dicarboxylic acid itaconate was also found to be an important feature of this microglial reactive state. Endogenous itaconate is synthesized by the enzyme aconitate decarboxylase 1 (referred to as IRG1) that is encoded by the *Acod1* gene (Michelucci et al. 2013). In turn, itaconate is a known activator of nuclear factor erythroid 2‐related factor 2 (NF2L2, also known as NRF2), a transcription factor that regulates the expression of genes related to antioxidant defenses and anti‐inflammatory processes, leading to an attenuation in reactive oxygen species (ROS) production and reduced toxicity (Mills et al. 2018). NF2L2/NRF2 protein is encoded by the *Nfe2l2* gene. In fact, our investigation revealed that an IRG1/itaconate/NF2L2 signaling axis functioned as a negative feedback loop, where endogenous levels of itaconate are involved in the regulated production of pro‐inflammatory cytokines in LPS‐treated microglia, while loss of NRF2 activation leads to increased pro‐inflammatory cytokine production resulting in increased ROS presence and neurotoxicity.

The results of this study provide deep and analytically reliable insights into the metabolic pathways and regulatory mechanisms governing microglial activation and immune responses within the CNS. By delineating the metabolic signatures associated with distinct activation states and immune stimuli, we aim to uncover potential therapeutic targets for neuroinflammatory disorders and neurological diseases characterized by dysregulated microglial function and metabolism.

## 2 Materials and Methods

### 2.1 Chemicals for Metabolic Profiling

Chloroform (LC–MS grade), ammonium formate (LC–MS grade), formic acid (FA) (99%, LC–MS grade), succinic acid (> 99%), malic acid (> 99%) and itaconic acid (> 99%) were obtained from Sigma‐Aldrich (Steinheim, Germany). Methanol (LC–MS grade) and acetonitrile (LC–MS grade) were purchased from Thermo Fisher Scientific (Zurich, Switzerland). The water was purified using a Milli‐Q Water system from MilliPore (Bedford, MA, USA).

### 2.2 Cell Culture and Treatment

BV2 mouse microglia (RRID: CVCL_0182) (passage 4, gift from Guy Brown, University of Cambridge) were cultivated in high glucose (25 mM) DMEM + glutamax medium (Gibco, Fisher Scientific, Waltham, MA, USA) supplemented with 10% FBS (fetal bovine serum) and 1% Penicillin/Streptomycin (P/S) and grown in an incubator at 37°C and 5% CO_2_. For experiments, the BV2 cells were seeded in 5% FBS DMEM. MN9D mouse dopaminergic neurons (RRID: CVCL_M067) (gift from Alfred Heller, University of Chicago) were cultured in DMEM/F12 (Gibco) under the same conditions as above. Cells were treated with 20 ng/mL interleukin‐4 (IL4, Pepro Tech) or 100 ng/mL LPS (from *Escherichia coli* , serotype 026: B6; Sigma Aldrich) for the indicated time. Cell lines were regularly tested for the absence of Mycoplasma using the LookOut Mycoplasma qPCR Detection Kit (Sigma Aldrich). For all experiments, BV2 cells were used at passage numbers ≤ 25. The BV2 and MN9D cell lines are not listed as commonly misidentified cell lines by the International Cell Line Authentication Committee (Capes‐Davis et al. 2010). BV2 cells are widely used in our laboratory, particularly in experiments investigating the expression of homeostatic microglial markers such as P2RY12 and TMEM119, as well as their response to LPS stimulation and subsequent induction of pro‐inflammatory markers including NOS2, TNFα, and IL1β. (Škandík et al. 2025) The MN9D neuronal cell line has been reported to maintain stable morphological characteristics over time and does not exhibit alterations in neurotoxic responses following co‐culture with BV2 cells and LPS treatment, as documented in previous studies. (Burguillos et al. 2011; Škandík et al. 2025; Friess et al. 2021).

### 2.3 Cell Sample Harvesting for Metabolic Profiling

Day one, cells were plated in 150 mm dishes, 2 × 10 (Tharp et al. 2024) cells per plate. A total of 30 dishes were prepared, each treatment (IL4, LPS, control (CTRL)) represented by 10 samples each. Day two, dishes were treated for 6 h according to the assigned treatment protocols (LPS, 100 ng/mL Sigma; IL4, 20 ng/mL Peprotech; CTRL, no treatment). Following incubation, cells were harvested on ice at approximately 95% confluence using MQ water. Medium was washed out, followed by washing of cells three times with cold PBS. Detachment of cells was performed using a rubber‐tipped cell scraper. Detached cells were collected in 5 mL cold MQ water and subsequently transferred to polypropylene tubes and snap‐frozen using liquid N_2_, followed by thawing at 37°C for 10 min. After repeating the freeze–thaw cycle twice, the cells were stored at −80°C until metabolite extraction.

### 2.4 Glial Cell Extraction and Following Microglia Isolation

Glial cells were isolated from the brains of female C57BL/6J mice (Charles River, Sulzfeld, Germany, stock #000664) on postnatal day 14–16. All the experimental procedures were carried out according to the European and Swedish animal welfare regulations approved by the northern Stockholm ethical committee (application nr. 13676‐2020) (*n* = 10 per preparation). Animals were deeply anesthetized with sodium pentobarbital (ABCUR AB, Sweden), and transcardially perfused with ice‐cold 1 × phosphate‐buffered saline without Ca^2+^ and Mg^2+^ (PBS; pH 7.4; GIBCO/Life Technologies #10010056). Brains were collected, the cerebella and olfactory bulbs were removed, and cerebra were placed into 50 mL tubes containing 1 × PBS and kept on ice. Briefly, dissected cerebra were transferred to Petri dishes and finely minced using scalpels. The tissue was then enzymatically digested in 2.5 mL of an enzyme solution consisting of 0.01% papain (0.1%; Roche #000000010108014001), 0.1% dispase II (Sigma‐Aldrich #D4693), 0.05% DNase I (Roche # 000000010104159001), and 12.4 mM MgSO₄ (Sigma‐Aldrich #M7506) made in 1× Hank's‐buffered salt solution (HBSS) without Ca^2+^ and Mg^2+^ (GIBCO/Life Technologies #14175095). Samples were incubated at 37°C for 10 min, after which the enzymatic activities were stopped with 20% cold heat‐inactivated fetal bovine serum (FBS; GIBCO/Life Technologies #10500064). Tissue homogenization was achieved by gentle trituration using a pipette until a uniform suspension was obtained. Cell suspensions were filtered through a 70 μm strainer, diluted with 20% FBS in HBSS, and centrifuged at 500 × *g* for 5 min at 4°C. After washing with 1× HBSS, cells were resuspended in 20% percoll solution (percoll plus, GE Healthcare, #GE17‐0891‐02); 10× phenol red HBSS (Gibco/Life Technologies #14060040) and 1× HBSS, and overlaid with an equal volume of 1× HBSS, and overlaid carefully with 1× HBSS, and centrifuged at 500 × *g* for 20 min at 4°C without brake to remove the myelin. Following removal of the supernatant, cells were washed in 1× HBSS and centrifuged again at 500 × *g* for 5 min at 4°C. The final pellet was resuspended in complete culture medium consisting of DMEM/F12 with Glutamax culture medium (Gibco/Life Technologies #31331028) and 10% heat‐inactivated FBS, supplemented with 10 ng/mL recombinant mouse M‐CSF (R&D Systems 416‐ML‐010). Cells were plated in a T75 flask and grown at 37°C in 5% CO_2_. The culture media were replaced every 2–3 days.

Primary microglia were isolated from mixed glial cultures after 10–14 days in vitro. Cells were washed with PBS and detached by incubation with 0.5% trypsin–EDTA (GIBCO/Life Technologies Cat#10779413) at 37°C for 10 min. Trypsin was neutralized with complete culture medium, and cells were collected into 50 mL tubes and centrifuged at 500 × *g* for 5 min at room temperature. Pellets were resuspended in a flow cytometry buffer (Biotechne, #FC001). Cells collected from all culture flasks were pooled and centrifuged as above. The cell pellet was incubated with CD11b microbeads (Miltenyi Biotec #130‐093‐636) for 10 min at 4°C for magnetic separation. Cells were washed with flow cytometry buffer and centrifuged again. Magnetic separation was performed using LS columns placed in a magnetic separator. Columns were pre‐equilibrated with FACS buffer, and the cell suspension was applied to the column. After washing with FACS buffer, columns were removed from the magnetic field, and CD11b‐positive microglia were eluted with complete culture medium using the plunger. Cell numbers and viability were determined using an automated cell counter.

### 2.5 Metabolite Extraction of the Polar Metabolome

Harvested cells were thawed at room temperature. Cellular debris was precipitated by centrifugation (10 min, 2200 RCF at 4°C) and removed by transferring a fixed volume of aqueous supernatant from each sample to fresh tubes. A pooled quality control (QC) sample was prepared by transferring an equal amount of cell extract from every sample to a single, fresh tube. A two‐phase system was obtained by the addition of chloroform (CHCl_3_) and methanol (MeOH) to a fixed proportion of 4:4:2.85 (CHCl_3_:MeOH:sample). Samples were gently vortexed and left to extract for 30 min at 4°C followed by centrifugation (10 min, 2200 RCF at 4°C). A fixed volume of aqueous supernatant was transferred from each sample to fresh tubes and evaporated at 45°C until dry. Samples were stored dry at −80°C until reconstituted in 75:25 acetonitrile and MilliQ‐water (AcN:MilliQ) before analysis. Each group of samples (IL4, LPS and CTRL) was thus represented by 10 technical replicates, each with one additional QC sample.

### 2.6 LC‐HRMS Analysis

Chomatographic separation was conducted on a UPLC Acquity (Waters, Manchester UK) coupled to a Synapt G2‐S mass spectrometer (Waters, Manchester UK). The LC was equipped with an AQUITY UPLC BEH Amide, 1.7 μm, 2.1 × 50 mm column (Waters). The software used for the LC‐HRMS analysis was MassLynx (Waters, V4.1). Mobile phase A consisted of 5 mM ammonium formate + 0.0125% FA in 95:5 AcN:Milli‐Q. Mobile phase B consisted of 5 mM ammonium formate + 0.0125% FA in 40:60 AcN:Milli‐Q. A nonlinear gradient was employed going from 100% A to 100% B within 17 min, after which the gradient returned to 100% A for 6 min to re‐equilibrate the column. The flow rate was set to 0.4 mL/min and the injection volume was 5 μL. Data acquisition stopped at 17 min before re‐equilibration of the column. The MS detector was set to a mass range of 50–800 Da. The source temperature was set to 120°C and the desolvation gas temperature was 500°C. Nitrogen was used both as desolvation gas with a flow rate of 1000 L/h and as cone gas with a flow rate of 50 L/h. The scan time was 0.3 s in centroid mode. Analysis was performed in resolution mode using negative and positive Electrospray ionization (ESI). Mass correction was done using a solution of 0.2 ng/mL Leucine‐Enkephalin (Leu‐Enk) in 1:1 AcN:Milli‐Q with 0.1% FA as lockspray with a scan time of 0.5 s and an interval of 30 s, using 3 scans to average and a mass window of ±0.5 Da.

System suitability, mass accuracy, and retention time drift were monitored by repeated injections of the QC sample prior to the analysis of the study‐specific samples. Moreover, the QC sample was injected at regular intervals throughout the analytical run to assess repeatability and overall system performance across the analytical batch.

### 2.7 Data Processing of LC–MS Data

Raw LC–MS data was extracted from Masslynx (Waters, V4.1) and converted into NetCDF files by Databridge (Waters, V4.1) to be used for further processing using the R‐package MetaboAnalystR (Pang et al. 2024). Data obtained in positive and negative ionization mode was processed separately. Firstly, a design of experiment (DOE) approach was used based on data obtained for the QC samples to optimize parameters for data processing as described by Pang et al. (2020). Peak picking, peak alignment, and gap filling were performed using the “centWave” algorithm along with the “loess” algorithm for peak alignment. Feature tables comprising detected features (retention time—Mass‐to‐Charge Ratio (*m/z*) pairs) and samples were thus generated for positive and negative ionization mode data. Only features with a retention time above 45 s and a molecular weight less than 800 Da were kept. These feature tables were subsequently imported into the online version of Metaboanalyst 5.0 in the subsection “Statistical Analysis”.

### 2.8 Visualization of Metabolic Alterations Caused by LPS and IL4 Treatment

Data filtering was performed according to technical repeatability in QC samples (Coefficient of Variation (CV) < 20%) followed by normalization to pooled samples from group QC samples (PQN), log transformation, and pareto scaling. Parametric One‐way Analysis of Variance (ANOVA) with post hoc tests (Tukey) was used to check for statistically significant features in regard to treatment. Furthermore, principal component analysis (PCA) and cluster analysis by heat maps were used to visualize group classification. The ability to classify the three sample groups (CTRL/LPS/IL4) through PCA was assessed through permutation analysis.

### 2.9 Selection and Annotation of Features Related to LPS and IL4 Treatment

Vulcano plots were used to select relevant features regarding metabolic changes caused by the treatments by LPS and IL4 in comparison to CTRL. The following criteria were employed: *p*‐value < 0.01, (corrected by the Benjamini–Hochberg procedure), fold‐change (FC) < 0.5 or > 1.5 as compared to CTRL. Furthermore, features were only retained in the data set if they were significant according to the post hoc tests performed in the ANOVA analysis as previously described.

Feature identification was performed based on database searches against the Human Metabolome Database (HMDB) (V 5.0) with a molecular weight tolerance of 10 ppm with the following adducts possible in positive ionization: M + H, M + H‐H_2_O, M + Na, M + K, M + 2Na‐H, and M + 2K‐H, while negative ionization utilized: M‐H, M‐H_2_O‐H, M + Cl, M + F, M + Na‐2H, and M + K‐2H. The selection of adducts was made according to known adduct formation patterns observed in the instrumentation used. The metabolites identified should be seen as putatively annotated compounds according to the Metabolomics Standards Initiative (MSI) nomenclature. The biological function of the metabolites was examined with a combined usage of the HMDB database together with the Small Molecular Pathway Database (SMPDB) to connect metabolites to biological pathways and identify interconnections between related pathways (Jewison et al. 2014).

### 2.10 Immunoblot Analysis

Total protein extracts were collected directly in 2.5 × Laemmli buffer containing Protease Inhibitor Cocktail (Roche) and PhosSTOP (Roche) using a cell scraper. Samples were sonicated (Diagenode, Bioruptor Pico) and boiled, and proteins were then separated by 10% Sodium Dodecyl Sulfate (SDS)–polyacrylamide gel electrophoresis (Mini‐Protean tetra Cell system (Bio‐Rad)) and blotted onto 0.45 μm pore‐size nitrocellulose membranes (Bio‐Rad) using the Mini Trans‐Blot wet transfer system (Bio‐Rad). Membranes were blocked in 5% milk (Semper) in PBS‐T 0.1% Tween (Sigma) in PBS (Santa Cruz Biotechnology) and washed three times with PBS‐T 0.1% Tween (Sigma) in PBS before incubating with primary antibodies. Primary antibodies anti‐Actin (Sigma ‐Aldrich, A3853) anti‐NRF2 (Cell Signaling Technology, 20 733), anti‐IRG1 (Abcam, 222411), anti‐NOS2 (Cell Signaling Technology, 13120), and anti‐Arg1 (Santa Cruz Biotechnology, sc‐18354) were diluted 1:1000 in 3% BSA in PBS‐T, and membranes were incubated with those antibodies overnight at 4°C. Membranes were washed three times with PBS‐T 0.1% Tween (Sigma) in PBS. Membranes were subsequently incubated with RDye secondary antibodies according to the manufacturer's instructions, then washed with PBS‐T before being visualized by the Odyssey CLx infrared imaging system (LI‐COR). Antibodies used in this study are summarized in Table S1. Obtained fluorescent signals were analyzed in Image Studio Lite, version 5.2 (LI‐COR) and densitometry was done in ImageJ, version 1.8.0. Levels of all analyzed proteins were normalized to Actin. The uncropped immunoblots can be found in Supporting Information.

### 2.11 Acod1 and Nfe2I2 Silencing by siRNA in BV2

BV2 cells were seeded in 6‐well plate dishes. Transfection was carried out 24 h after plating either with nontargeting siRNA (NTC, 40 nM, used as control), siRNA against *Acod1* (40 nM) or siRNA against *Nfe2I2* (40 nM) with 3 μL of Lipofectamine 3000 (Invitrogen) respectively. Nontargeting control (NTC, D‐001810) and *Acod1* (*L‐048308*) or *Nfe2I2* (*L‐048308*) ON‐TARGET plus SMARTpools siRNAs were obtained from Dharmacon (Dharmacon, Lafayette, CO, USA). The sequences of the ON‐TARGET plus SMARTpools siRNAs used in this study can be found in Table S2. Cells were harvested after the indicated treatment time.

### 2.12 Acod1 Silencing by siRNA in BV2 Murine Primary Microglia

Primary microglia were transfected with siRNA using the Glial‐Mag transfection reagent and magnetic plate system (OZ Biosciences) according to the manufacturer's protocol with minor modifications. The procedure was performed in either 12‐well or 96‐well plate formats, with reagent volumes adjusted accordingly. Briefly, siRNA (20 μM stock concentration) was diluted in DMEM/F12 without serum or antibiotics to a final volume of 255 μL per well (12‐well) or 20 μL per well (96‐well). Specifically, 1 μL (12‐well) or 0.1 μL (96‐well) of siRNA was mixed by vortexing with the respective volume of DMEM/F12. This solution was then combined with 0.6 μL (12‐well) or 0.06 μL (96‐well) of Glial‐Mag reagent and mixed gently by pipetting up and down 4–5 times. The mixture was incubated at room temperature for 20 min to allow complex formation. Meanwhile, the culture medium in each well was replaced with fresh DMEM/F12 without serum or antibiotics (675 μL for 12‐well or 80 μL for 96‐well). Following incubation, the siRNA–Glial‐Mag complex was added dropwise to each well along with 8.5 μL (12‐well) or 1 μL (96‐well) of Glial‐Boost (100×), as recommended in the kit protocol. The culture plates were then placed on the magnetic plate inside a humidified incubator at 37°C with 5% CO_2_ for 30 min to facilitate magnetic‐assisted transfection. After removing the magnetic plate, cells were incubated for an additional 3 h under the same conditions. Subsequently, the medium was replaced with complete culture medium (DMEM/F12 supplemented with 5% FBS and 1% P/S), and cells were allowed to recover for 24 h prior to downstream experimental procedures.

### 2.13 RNA Isolation, cDNA Synthesis and qPCR

Total RNA was isolated using the RNeasy Plus Mini kit (Qiagen) following the manufacturer's instructions. The yield and quality of the isolated mRNA were analyzed using a NanoDrop spectrophotometer (ThermoFisher Scientific). The cDNA was synthesized from 1 μg of RNA using Oligo dT, dNTPs, and Superscript IV (Invitrogen) with a Bio‐Rad T100 PCR Thermal Cycler. RT‐qPCR was performed using SSoAdvanced Universal SYBR Green Supermix (Bio‐Rad) and run on a CFX Duet Real‐Time PCR System (Bio‐Rad). Results were calculated using the delta Ct method and represented as a fold over control cells. The sequences of the pre‐designed primers (KiCqStart Primers, Sigma) used in this study can be found in Table S3.

### 2.14 Multiplex Cytokine Measurement

BV2 cells were transfected accordingly, and conditioned medium was collected from untreated and LPS 24 h, treated BV2. Cytokine levels were determined using the LEGENDplex MU Cytokine Release Syndrome Panel (13‐plex) w/VbP (Biolegend, 741024) according to the manufacturer's guidelines. In brief, the assay was carried out in a 96‐well plate using flow cytometry (BD FACSCanto II) and the following cytokines were measured: CCL2 (MCP‐1), IL10, CCL4 (MIP‐1β), IFN‐α, CXCL9 (MIG), CXCL10 (IP‐10), TNF‐α, IL6, VEGF, IL4, CCL3 (MIP‐1α), IFN‐γ, and GM‐CSF.

### 2.15 Mitochondrial Superoxide Assay

BV2 cells were transfected and treated with LPS for 24 h. Cells were washed with HBSS and then stained with the MitoSox Red Flow Cytometry Assay Kit (5 μM, Fisher Scientific) for 20 min at 37°C. After staining, the cells were washed with HBSS and analyzed by flow cytometry. As a positive control for mitochondrial derived ROS (mtROS) production, the cells were incubated with Antimycin A (10 μM) for 20 min before staining with MitoSox. The BD FACSCanto II flow cytometry system was used, and data were analyzed using FlowJo v10.8.1.

### 2.16 Neurotoxicity Assay

MN9D neurons were stained with 2.5 μM CellTracker Green CMFDA (Invitrogen) for 30 min at 37°C. BV2 were plated, and the next day transfected. After 24 h transfection, the BV2 were plated as a direct co‐culture with MN9D. After sufficient time when BV2 cells were fully attached to the cell plate surface, LPS treatment for 24 h was performed. Prior to imaging, the cells were stained with 0.1 mg/mL Hoechst 33342 (Invitrogen) for 10 min at 37°C. At least 6 pictures were taken, and a minimum of 100 MN9D cells were counted per independent replicate by inverted fluorescent microscope Axio Observer 3 (ZEISS). The number of dying cells was measured quantitatively by assessing the percentage of cells with fragmented, damaged, or condensed nuclei. The effect of the fluorescent probe and LPS treatment itself on MN9D monoculture is presented in Figure S1.

### 2.17 Extracellular Flux Analysis (Seahorse)

For the long recording experiments, the day before the experiment, BV2 microglia were seeded in a 24‐well plate format in grown media DMEM, 25 mM glucose, 1 mM pyruvate, 2 mM glutamax, and 5% FBS. For primary microglia, cells were seeded in a 96XF plate in DMEM/F12 supplemented with 5% FBS and 1% P/S. The medium was changed to DMEM with the same components (XF DMEM base) as above without buffer bicarbonate and 5% FBS and left for 1 h in a non‐CO_2_ incubator. After that, the Oxygen Consumption Rate (OCR) and Extracellular Acidification Rate (ECAR) rates were acquired, and after baseline recording, the injection of LPS and IL4 was performed. OCR and ECAR were recorded every 20 min to avoid useless stress to the cells during the mixing command. As control, the injection of PBS in control or silenced cells (siNTC, used as control) or siAcod1 was performed, and after some h of recording, the injection of oligomycin (10 μM), FCCP (10 μM) and rotenone/antimycin (15 μM) was performed to control the validity of the assay. The measurement was done using a Seahorse Biosciences XF24 Analyzer for BV2 and XF96 for primary microglia.

For the Mito Stress assay, BV2 cells were seeded at 10 000 cells/well 1 day prior to the assay, and primary microglial cells at 30000 cells/well 2 days prior. Where applicable, siRNA‐mediated silencing was performed one day before the assay in 96‐well Seahorse plates. XF96 Cell Culture Microplates (Agilent, Santa Clara, CA, USA). On the day of the analysis, assay media was prepared as above, and the metabolic flux was measured at basal level after injection of a final concentration of 1 μM oligomycin, FCCP 3 + 2 μM for primary glia and 1.5–2.5 μM for BV2, and a 1:1 mixture of rotenone and antimycin A at a final concentration of 4 μM each.

Data was normalized based on the number of cells per well. Cell number quantification was done by staining nuclei with Hoescht 33342 (Molecular Probes, Thermo Fisher Scientific) for 10 min and then imaging each well using BD pathway 855 (BD Biosciences, Franklin Lakes, USA) with a 20× objective and a montage 5 × 4. Cell numbers were counted using the CellProfiler software.

To assess whether in vitro culture conditions using DMEM + glutamax with high glucose (25 mM) influence BV2 microglial metabolism compared to a more physiological glucose concentration (5.5 mM), we performed an initial optimization experiment. The BV2 microglia were pre‐stimulated for 6 h with either LPS or IL4, and their metabolic responses were measured immediately thereafter. Data from the two glucose conditions indicate that lower glucose availability alters cellular metabolism, with BV2 cells increasing their respiratory rate and relying less on glucose for energy production. However, in both high‐ and low‐glucose conditions, IL4 treatment produced minimal to no metabolic changes, whereas LPS stimulation increased the ECAR levels and reduced respiratory rate in both cases. Thus, the observed metabolic shifts are LPS‐dependent and not attributable to glucose concentration (Figure S2A,B).

### 2.18 ELISA Measurement

BV2 microglia were transfected accordingly, and conditioned medium was collected from untreated and LPS (24 h) treated BV2. IL1β levels were determined using Mouse IL1β ELISA (Invitrogen, 18061673) according to the manufacturer's guidelines. In brief, the assay was carried out in a 96‐well plate using a Tecan Spark plate reader (Tecan).

### 2.19 Quantification and Statistical Analysis

The sample size for each experiment was not pre‐determined by formal calculation (G*power) but was based on previous experimental setups using BV2 microglia, MN9D neurons, or primary microglial cultures (Škandík et al. 2025; Stratoulias et al. 2023; Grabert et al. 2023). Technical replicates (wells, Petri dishes, pooled animals) are denoted as *n*, and biological replicates (independent passages or experimental runs) as *N*. The corresponding *n/N* values are provided in related figure legends. No outliers were detected using the ROUT method (*Q* = 1%). Given the limited sample size, tests for normality (Shapiro–Wilk) were not conducted; data were assumed to follow a Gaussian distribution, and parametric analyses were applied.

Statistical analyses were performed using GraphPad Prism v10 software. Parametric one‐way ANOVA with post hoc tests (Tukey HSD, *p*‐value < 0.01) (corrected by the Benjamini‐Hochberg procedure) and FC < 0.5 or > 1.5 were used to identify statistically significant features from LC‐HRMS data regarding treatment effects. A more detailed description of statistical analyses including sample size, tests used, and multiple comparison conditions for each experiment can be found in Table S4. A *p*‐value of less than 0.05 was considered statistically significant.

## 3 Results

### 3.1 LC‐HRMS Analysis of the Polar Metabolome Reveals Distinct Features for the Pro‐Inflammatory and Anti‐Inflammatory Microglial Reactive States

**FIGURE 1:**
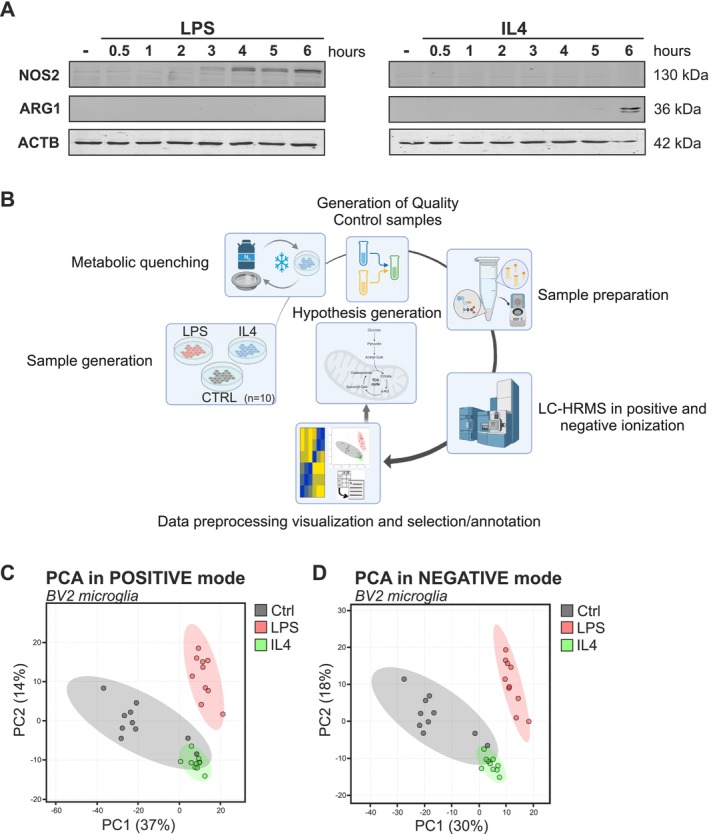
Metabolic profiling and PCA analysis of the polar metabolome of LPS‐ and IL4‐activated microglia. (A) Western blot analysis demonstrating protein expression of NOS2, ARG1 and ACTB in BV2 microglia treated with LPS (100 ng/mL) or IL4 (20 ng/mL) in time course matter from 30 min to 6 h. Control cells are treated with PBS for 6 h. (B) Workflow illustrates the key steps in profiling polar metabolites across three conditions: CTRL cells, LPS‐treated cells (6 h), and IL4‐treated microglia (6 h). After sample generation, rapid metabolic quenching was performed using liquid nitrogen to halt cellular activity, followed by PBS washes to remove residual growth medium. A Quality Control (QC) sample was prepared by pooling aliquots from all samples to ensure consistency throughout the analysis. Polar metabolites were extracted using a methanol‐chloroform (1:1) mixture. LC‐HRMS analysis was then conducted in both positive and negative ion modes with MS^E^ acquisition, incorporating regular QC sample injections to monitor and correct for retention time variation. Data processing involved feature selection and visualization with Metaboanalyst, followed by metabolite annotation via database matching (HMDB) and comparison with standards. Functional insights into the metabolic data were derived through integrated database searches, enabling hypothesis generation on the biological roles of identified metabolites. (C, D) PCA plots represent three different clusters of samples based on their similarities in positive and negative mode. The *p*‐values reported with the PCAs are (based on 999 permutations): 0.001 for both ionization modes.

While metabolic changes associated with peripheral macrophage activation have been explored extensively, less attention has been given to the CNS resident microglia (Lauro and Limatola 2020; Liu et al. 2021). In this study, LPS‐ or IL4‐stimulated BV2 microglial cells were used to explore metabolic changes associated with the pro‐inflammatory and the anti‐inflammatory microglial reactive states, respectively (Zuiderwijk‐Sick et al. 2021; Burguillos et al. 2011). As shown in Figure 1A, LPS‐treated BV2 microglia exhibited, within 3 h, visible expression of nitric oxide synthase 2 (NOS2, also known as inducible nitric oxide synthase, iNOS), a recognized marker of the pro‐inflammatory microglial reactive state. Changes in protein expression levels of arginase 1 (ARG1), a marker for IL4‐mediated anti‐inflammatory responses, became apparent after 5 h of treatment. Within the time frames investigated, responses to both treatments appeared to be fully present at the 6‐h time point. Moreover, the treatments produced two distinct microglial responses, as NOS2 was not detectable in IL4‐treated microglia, and ARG1 expression was absent in LPS‐treated microglia across all observed time points. Based on these findings, we selected a 6‐h treatment duration for both LPS and IL4 as the optimal condition for subsequent in‐depth metabolic profiling experiments.

The workflow for metabolic profiling of the polar metabolome employed is presented in Figure 1B. In brief, sample generation includes 10 technical replicates of untreated microglia cells (CTRL), 6 h LPS‐treated BV2 microglia, and 6 h IL4‐treated BV2 microglia. Rapid metabolic quenching was achieved by snap freezing the samples in liquid N_2_ followed by thawing at 37°C; this procedure was repeated twice. Prior to sample preparation with liquid–liquid extraction (LLE), a QC sample was created by pooling equal volumes from each of the study samples. Each sample was extracted with a mixture of MeOH and CHCl_3_ (1:1) to isolate the polar metabolites. Prior to analyzing the study samples, the system was pre‐conditioned by repeated injections of QC samples to minimize retention time drift (Engskog et al. 2016). LC‐HRMS analysis was performed in both positive and negative ionization (resolution mode) with MS^E^, which included re‐occurring QC sample injections throughout the analytical runs. The obtained data was pre‐processed with MetaboanalystR (Pang et al. 2024) to generate feature tables for visualization and feature selection by the online software Metaboanalyst 5.0 (Pang et al. 2021, 2022). Annotation was performed by searches against the HMDB database (Wishart et al. 2022) as well as comparisons with analytical standards and in‐house databases. Biological function was examined through the combined use of HMDB and SMPDB (Jewison et al. 2014; Wishart et al. 2022; Frolkis et al. 2010).

**FIGURE 2:**
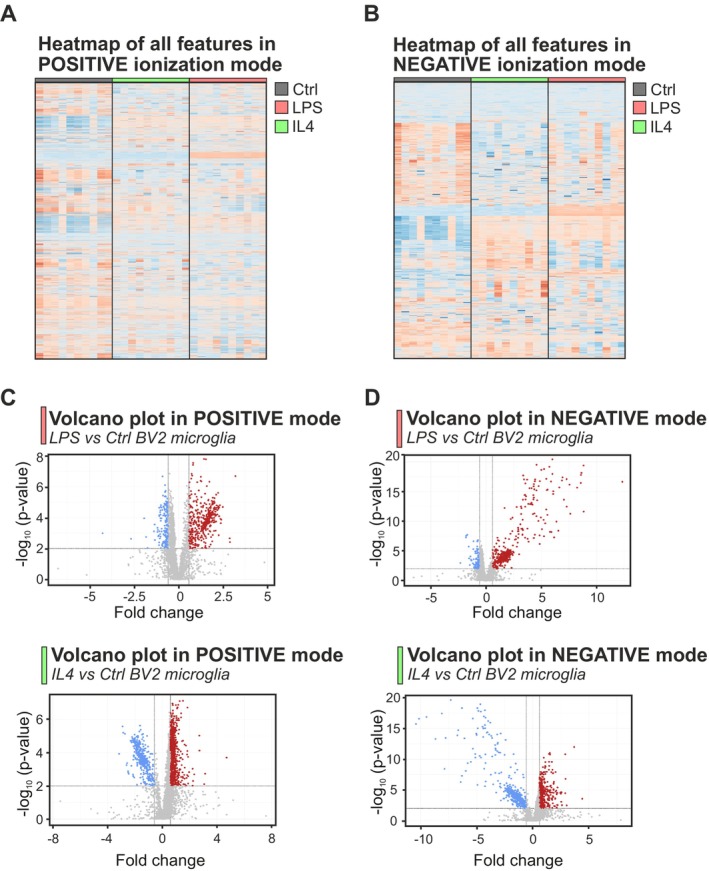
Visualization of metabolic changes in the polar metabolome across control, LPS‐treated, and IL4‐treated BV2 microglia. (A, B) Heatmap representations of metabolomic features obtained in positive and negative ionization modes, showing relative feature intensities across samples. Each column represents an individual sample, with color gradients indicating variations in feature abundance. The heatmaps reveal distinct clustering of features based on treatment groups, highlighting treatment‐specific metabolic changes. (C, D) Volcano plots depicting relation between metabolite fold changes and their statistical significance between LPS/IL4 and CTRL for both positive and negative ionization modes. Each point represents a metabolite feature, with red and blue points indicating up‐ and down‐regulated features, respectively, based on *p*‐value and fold‐change thresholds. Data are from *n* = 10 Petri dishes for CTRL, LPS (100 ng/mL, 6 h), or IL4 (20 ng/mL, 6 h)‐treated groups from the same passage *N* = 1.

From the LC‐HRMS analysis, we detected 4614 and 4489 features in positive and negative ionization modes, respectively. Features represent unique combinations of *m/z* and retention times. A refined set of 49 known metabolites was annotated and analyzed, representing key pathways such as glycolysis, the Tricarboxylic Acid Cycle (TCA), Pentose Phosphate Pathway (PPP), amino acid metabolism, and redox regulation. To visualize whether LPS and IL4 treatments had different effects on the microglial polar metabolome, the data sets were first filtered based on two criteria: (i) features must demonstrate a retention time above 45 s and (ii) a CV < 20% in the QCs in order to be deemed stable for further analysis. 3060 (out of 4614) and 2850 (out of 4489) of the features in positive and negative ionization modes, respectively, fulfilled these criteria and were thus considered analytically valid for further processing (Figure S3). PCA models, as well as volcano plots and heatmap representations of all features for these two data sets, are depicted in Figures 1C,D and 2A–D. The PCA models demonstrated that each sample group forms its own cluster, thus indicating a significant change (*p*‐values reported with the PCAs are (based on 999 permutations): 0.001 for both ionization modes) in the polar metabolome between those sample groups (Figure 1C,D). The heatmap representations visualize changes as relative feature concentrations between sample groups, as well as visualize the technical and biological variation between the sample groups and intra‐group variations (Figure 2A,B).

In summary, treatments with LPS and IL4 resulted in significantly altered polar metabolome profiles.

### 3.2 Differential Alterations in Metabolites Linked to Glycolysis, PPP, and TCA Cycle Are Observed in LPS‐ Versus IL4‐Treated Microglia

**FIGURE 3:**
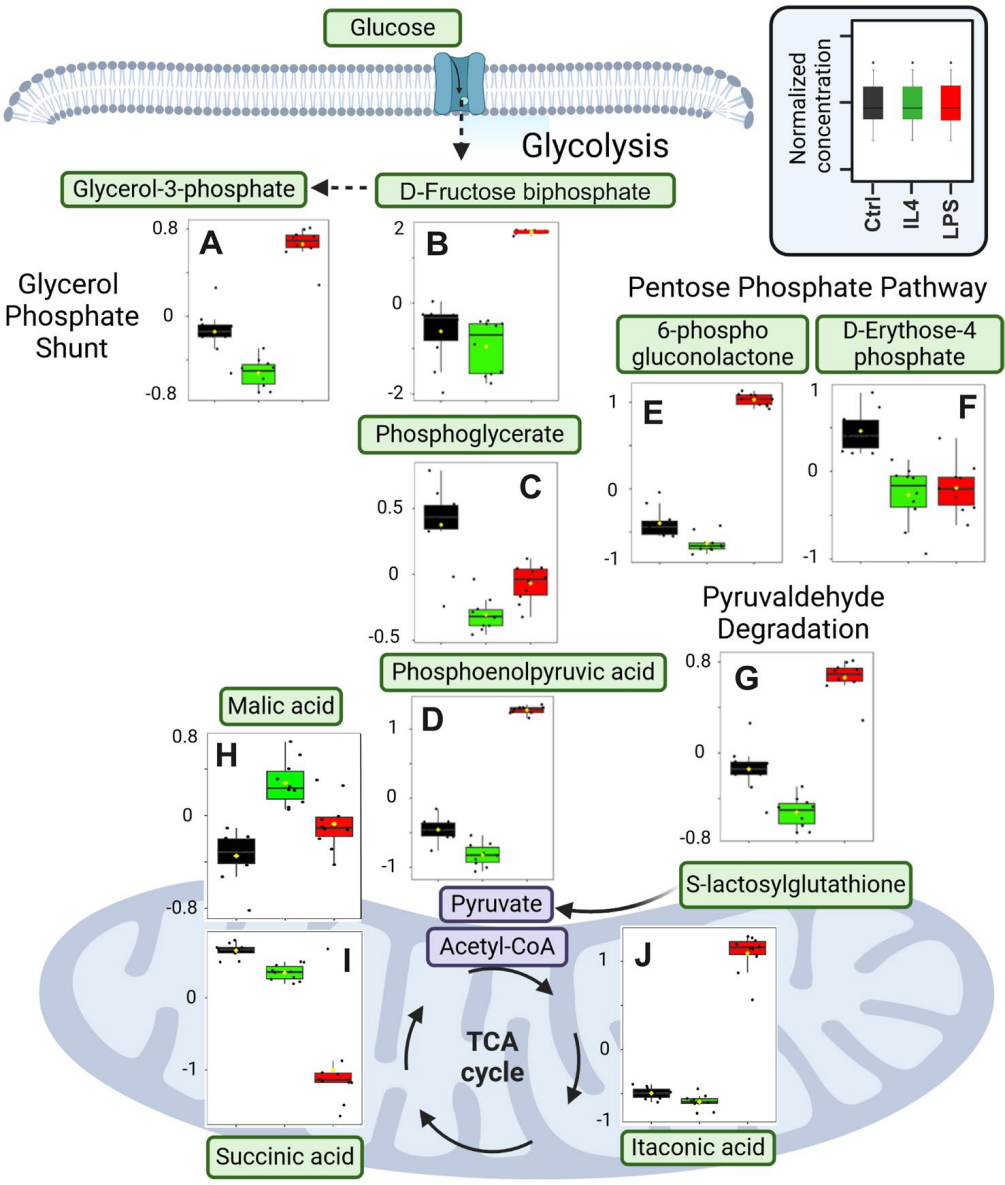
Overview of significant microglial metabolic alterations in glycolysis, PPP, glycerol phosphate shunt, and TCA cycle in response to LPS and IL4 treatments. Graphs represent relative concentrations of the following metabolites: (A) Glycerol‐3‐phosphate (glycerol phosphate shunt); (B) d‐Fructose bisphosphate (glycolysis); (C) Phosphoglycerate (glycolysis); (D) Phosphoenolpyruvic acid (glycolysis); (E) 6‐Phosphogluconolactone (pentose phosphate pathway, PPP); (F) D‐Erythose‐4‐phosphate (PPP); (G) S‐lactosylglutathione (pyruvaldehyde degradation); (H) Malic acid (tricarboxylic acid (TCA) cycle); (I) Succinic acid (TCA cycle); (J) Itaconic acid (TCA cycle). Data are from *n* = 10 Petri dishes, LPS (100 ng/mL, 6 h), or IL4 (20 ng/mL, 6 h)‐treated groups from the same passage *N* = 1.

Features showing significant differences in abundance after treatment with LPS or IL4 (*p* < 0.01 and fold change [FC] < 0.5 or > 1.5) were selected from the Volcano plots (Figure 2C,D). These features fall into three categories based on their relative concentration compared to CTRL cells: (i) up‐regulated features (FC > 1.5), (ii) down‐regulated features (FC < 0.5), and (iii) treatment‐specific anti‐correlated features, which display opposing regulation between LPS and IL4 (e.g., increased by LPS and decreased by IL4, or vice versa). These categories form the basis for further analysis of differential metabolic responses to pro‐ versus anti‐inflammatory stimulation. Annotation of metabolites was performed through the usage of the HMDB database (Wishart et al. 2022) (10 ppm threshold), all in accordance with the guidelines provided by the MSI, along with comparison to analytical standards and in‐house databases when available. In summary, 85 significant metabolites were annotated regarding LPS treatment of microglia, while 89 metabolites related to IL4 treatment (Figure S3). These metabolites relate to a wide range of substance classes, spanning from smaller amino‐ and organic acids to larger polar lipid classes and carnitines. Specifically, several of these metabolites are found within the central carbon metabolism, that is, glycolysis, PPP, as well as downstream metabolic pathways, such as the TCA cycle (Figure 3A–J). Furthermore, some of these metabolites belong to the carnitine shuttling system and hence relate to fatty acid β‐oxidation (Figure S4A–I).

Regarding glycolysis, a major up‐regulation in LPS‐treated microglia was observed for d‐Fructose‐bisphosphate (FC > 40), a change not observed in IL4‐treated microglia (Figure 3B). Phosphoenolpyruvic acid demonstrated an anti‐correlation, being up‐regulated in LPS‐treated microglia (FC = 39.9) but down‐regulated in IL4‐treated microglia (FC = 0.4) (Figure 3D). Moreover, Phosphoglycerate displayed a down‐regulation in both treatments (FC = 0.4) (Figure 3C). Furthermore, the interconnected glycerol phosphate shunt was represented by the up‐regulation of glycerol‐3‐phosphate in LPS‐treated cells (FC = 1.8), a change not observed upon IL4 treatment (Figure 3A). S‐lactosylglutathione, situated in the pyruvaldehyde degradation pathway, displayed an anti‐correlation with an up‐regulation in LPS samples (FC = 2.6) and a tendency toward down‐regulation in IL4 samples (FC = 0.6) (Figure 3G). With regard to the interconnection to PPP, the following significant metabolic changes were observed: d‐Erythose‐4‐phosphate displayed down‐regulation in both treatments as compared to control cells (FC = 0.5) while 6‐phosphogluconolactone displayed anti‐correlation (LPS; FC = 11.5 IL4; FC = 0.6) (Figure 3E,F).

Significantly affected metabolites in the TCA cycle included itaconic acid exhibiting an anti‐correlation (LPS; FC = 18.6; IL4; FC = 0.5), malic acid with an up‐regulation in IL4‐treated microglia (FC = 1.5) and succinic acid showing a down‐regulation in both treatments (FC = 0.1) (Figure 3H,I).

Several other metabolites were found to be significantly altered in microglia upon treatment with either LPS or IL4. Of particular importance here are changes related to fatty acid β‐oxidation and the route through the carnitine shuttle system responsible for the transport of fatty acids across the inner mitochondrial membrane (Figure S4A–I) (Houten et al. 2016). Increased presences were observed for several long and medium chain acylcarnitines (3‐hydroxyhexadecanoylcarnitine, 3‐methylglutarylcarnitine and cervonyl carnitine) in both LPS‐ and IL4‐treated microglia (Figure S4C–E). LPS‐treated microglia also showed a significant up‐regulation of l‐acetylcarnitine, while l‐carnitine was also found to be down‐regulated in both treatments (Figure S4A,B). Moreover, down‐regulation was observed in LPS‐treated microglia for the following short chain acylcarnitines: tiglylcarnitine, isobutyryl‐L‐carnitine, and propionylcarnitine, while l‐carnitine was also found to be down‐regulated (Figure S4F–I).

Collectively, the above metabolomic data strongly suggest that LPS treatment, as well as IL4 treatment, leads to distinct energy‐related metabolic changes that could contribute to the observed microglial reactive states.

### 3.3 LPS Treatment in Microglia Indicates an Increase in Aerobic Glycolysis

**FIGURE 4:**
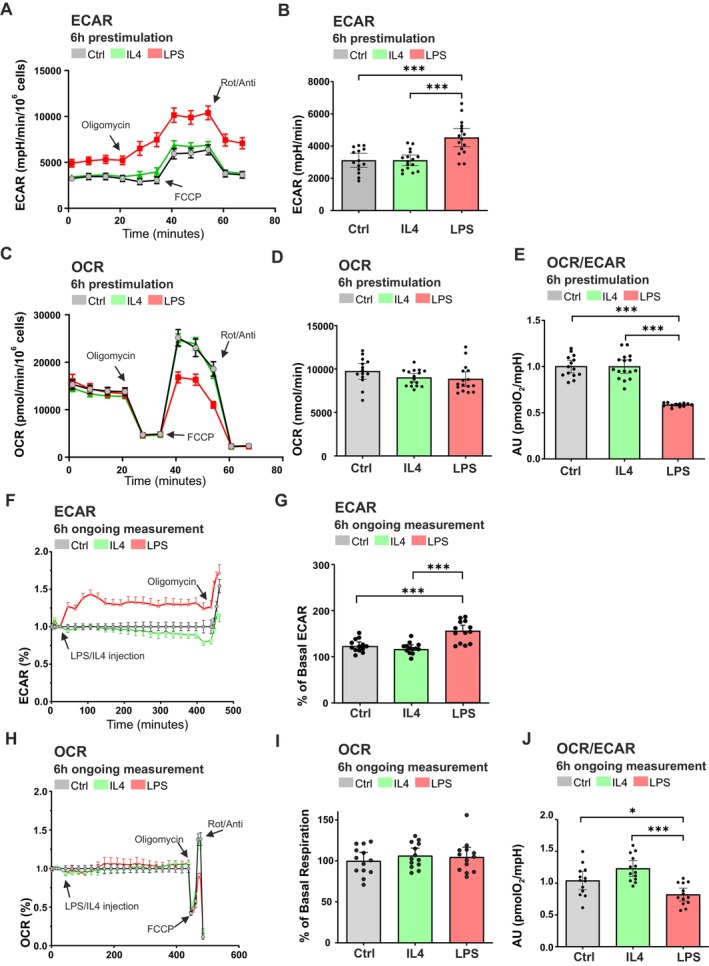
LPS treatment shifts microglial metabolism from oxidative phosphorylation to glycolysis. Two experimental approaches employing the Seahorse metabolic analyzer were used to study the effect of IL4 (20 ng/mL) and LPS (100 ng/mL) treatment in BV2 microglia. Panels A–E show 6‐h long treatment at standard culture conditions with subsequent metabolic analysis representing (A) ECAR analysis curve with (B) ECAR quantification, (C) OCR analysis curve with (D) quantification, and (E) OCR/ECAR ratio. Panels F–J show results from a second experimental approach using BV2 cells treated with the same concentrations of IL‐4 and LPS, measured continuously over 6 h using the Seahorse analyzer. Panel (F) shows representative ECAR analysis curve over 6‐h long measurement with (G) quantification, (H) OCR analysis curve with (I) quantification, and (J) OCR/ECAR ratio for 6‐h long ongoing measurement in the Seahorse analyzer. Data are mean ± SEM from *n =* 16 independent points of measurement from *N* = 2 different passages (A–E); *n =* 13 points of measurement from *N =* 2 different passages (F–J). Statistical annotations *p < 0.05; **p < 0.01; ***p < 0.001; for the indicated comparison. More detailed information about sample size and used statistical tests can be found in Table S4.

A Seahorse real‐time extracellular flux analysis was performed to further investigate how energy is generated through glycolysis and/or mitochondrial respiration in each microglial activation state. Substrate utilization was assessed by measuring ECAR, which reflects glycolytic activity through lactate production as a main contributor to extracellular acidification, and OCR, representing mitochondrial respiration through oxidative phosphorylation (Figure 4). Two experimental approaches were employed: (i) BV2 microglia were treated with LPS and IL4 for 6 h under standard culture conditions prior to metabolic measurement using the Seahorse Analyzer (Figure 4A–E); and (ii) BV2 microglia were exposed to LPS and IL4 during a real‐time 6‐h Seahorse assay to monitor dynamic metabolic responses throughout the treatment period (Figure 4F–J). In the pre‐treatment experimental approach, LPS stimulation led to a significant increase in basal ECAR compared to untreated control and IL4‐treated microglia, indicating enhanced glycolytic activity and a metabolic shift toward aerobic glycolysis through increased lactate metabolism (Figure 4A,B). Upon the addition of oligomycin, an inhibitor of ATP‐linked mitochondrial respiration, LPS‐treated microglia showed a further increase in ECAR (Figure S5A), confirming enhanced reliance on glycolysis for ATP production. OCR was measured to evaluate mitochondrial respiration in LPS‐ and IL4‐treated BV2 microglia with no significant changes observed in OCR after LPS or IL4 treatment following oligomycin administration (Figure 4C,D). However, OCR measured after the addition of oligomycin resulted in a significant decrease in BV2 microglia treated with LPS and IL4 (Figure S5B). Maximal respiration capacity, assessed by the addition of FCCP to uncouple ATP synthesis from mitochondrial respiration, was significantly reduced in LPS‐treated microglia compared to both control and IL4‐treated cells (Figure S5C). To further investigate the metabolic impact of LPS stimulation, we assessed the OCR to ECAR ratio and found a significant reduction in LPS‐treated microglia, consistent with a shift toward glycolytic metabolism (Figure 4E). Data originating from the second experimental approach in which LPS and IL4 were applied during the Seahorse assay (Figure 4F–J) confirm an initial burst in ECAR and glycolytic metabolism after LPS administration (Figure 4F). The following quantification confirms ECAR up‐regulation at the basal state (Figure 4B) and after oligomycin addition (Figure S5D) compared to IL4 treatment or untreated cells. OCR measurement at the basal state, after oligomycin, and FCCP addition reinforce the measurement of the first experimental approach (Figure 4H,I; Figure S5E,F). These findings demonstrate that LPS stimulation induces a rapid metabolic reprogramming in microglia from the early time of treatment that sustained the whole 6‐h long measurement, characterized by increased glycolytic flux. In contrast, IL4‐treated microglia show minimal changes in ECAR and OCR compared to untreated cells. The consistent results obtained with both experimental approaches, (i) pre‐stimulation and (ii) treatment during the Seahorse assay, underscore the robustness of these metabolic shifts associated with distinct microglial activation states.

### 3.4 An IRG1/Itaconate/NF2L2 Signaling Axis Regulates the LPS‐Induced Microglia Pro‐Inflammatory Activation State

Itaconate is one of the most intriguing metabolites that displayed a noteworthy difference in response between LPS‐ and IL4‐treated microglia. Itaconate is a critical mediator of metabolic changes as demonstrated in several studies and is produced through the conversion of aconitate by IRG1 (O'Neill and Artyomov 2019). Itaconate is a known activator of NF2L2/NRF2, a transcription factor, primarily through the alkylation of cysteine residues on Kelch‐like ECH‐associated protein 1 (KEAP1). KEAP1 forms part of an E3 ubiquitin ligase, which regulates the activity of NF2L2/NRF2 by targeting it for ubiquitination and proteasome‐dependent degradation (Ahmed et al. 2017; Itoh et al. 2010). Itaconate‐mediated KEAP1 modification facilitates the release of NF2L2/NRF2, its translocation to the nucleus, and the regulation of genes related to antioxidant defenses as well as anti‐inflammatory processes, ultimately leading to a reduction in cellular reactive oxygen species and inflammation.

**FIGURE 5:**
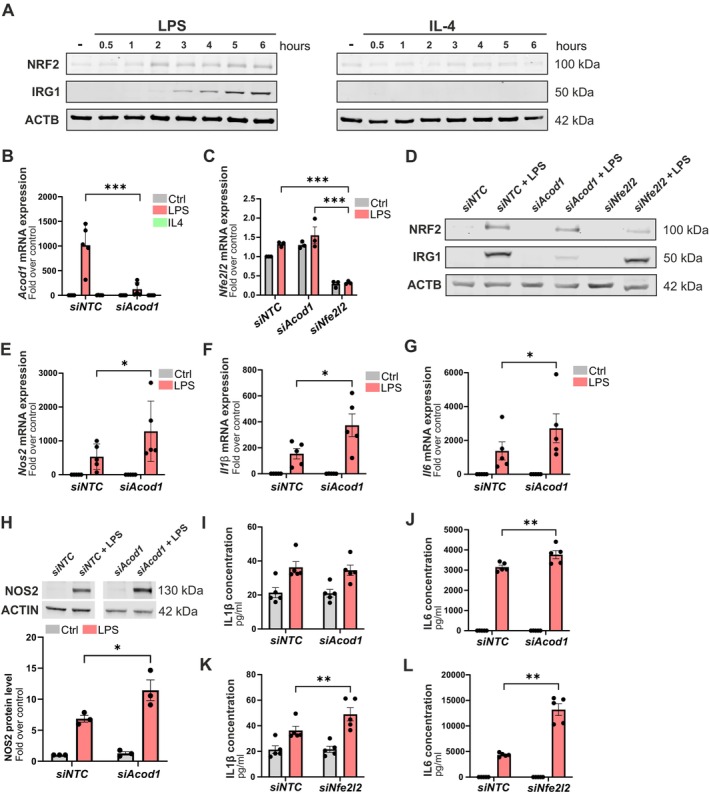
An IRG1‐itaconate‐NF2L2 axis regulates LPS‐induced pro‐inflammatory reactivity in microglia. (A) Western blot analysis of IRG1, NF2L2/NRF2, and ACTB protein expressions in BV2 microglia treated with LPS and IL4 for 6 h. Control cells are treated with PBS for 6 h. (B) RT‐qPCR analysis of *Acod1* expression in BV2 microglia transfected with siRNAs pool targeting *Acod1* (*siAcod1*) or siRNA nontargeting control (*siNTC*). (C) RT‐qPCR analysis of *Nfe2l2* expression in BV2 microglia transfected with *siAcod1*, *siNfe2l2*, or *siNTC*. (D) Western blot analysis of IRG1, NF2L2/NRF2, and ACTB protein expressions in LPS‐treated BV2 microglia transfected with *siAcod1*, *siNfe2l2*, or *siNTC*. (E–G) RT‐qPCR analysis of *Nos2, Il1β* and *Il6* gene expression in LPS‐treated BV2 microglia transfected with *siAcod1* or *siNTC*. (H) Western blot analysis of NOS2 and ACTB protein expressions in 24 h LPS‐treated BV2 microglia transfected with *siAcod1* or *siNTC*. Graph depicts quantification of NOS2 expression as a ratio to ACTB expression. (I) Secreted IL1β expression levels measured by ELISA in supernatant of 24 h LPS‐treated BV2 microglia transfected with *siAcod1* or *siNTC*. (J) Secreted IL6 expression levels measured by flow cytometry multi‐ELISA LEGENDplex in supernatant of 24 h LPS‐treated BV2 microglia transfected with *siAcod1* or *siNTC*. (K) Secreted IL1β expression levels measured by ELISA of 24 h LPS‐treated BV2 microglia transfected with *siNfe2l2* or *siNTC*. (L) Secreted IL6 expression levels measured by flow cytometry multi‐ELISA LEGENDplex in 24 h LPS‐treated BV2 microglia transfected with *siNfe2l2* or *siNTC*. Data are mean ± SEM from multiple independent biological replicates (passages) *N =* 5 (B, E, F, G, I, J, K, L) and *N =* 3 (A, C, D, H) for untreated, LPS (100 ng/mL), or IL4 (20 ng/mL)‐treated groups. Statistical annotations *p < 0.05; **p < 0.01; ***p < 0.001; for the indicated comparison. More detailed information about sample size and statistical tests can be found in Table S4.

To further investigate a possible role for the itaconate‐dependent signaling pathway in mediating microglial pro‐inflammatory activation, protein expression levels of IRG1 and NF2L2/NRF2 were assessed in LPS‐ and IL4‐treated microglia. Western blot analysis demonstrated a significant up‐regulation of both IRG1 and NF2L2/NRF2 protein expression levels in LPS‐treated microglia, but not in IL4‐treated microglia, as compared to untreated controls (Figure 5A). Hence, an IRG1/itaconate/NRF2 signaling axis appears to specifically contribute to the LPS‐induced activation of microglia.

To get further insight into the importance of the IRG1/itaconate/NF2L2 signaling axis in the regulation of the acquisition of a pro‐inflammatory microglial reactive state induced by LPS, the expression of its upstream component, i.e., IRG1, was reduced taking advantage of a small interfering RNA pool targeting *Acod1* gene expression. The efficiency of the siRNA‐mediated repression in *Acod1* gene expression in BV2 microglia is shown at both messenger and protein levels in Figure 5B,D. Thereafter, *Acod1*‐siRNA and siRNA‐nontargeting control (*siNTC*) transfected BV2 microglia were exposed to LPS. RT‐qPCR analysis revealed that the mRNA expression levels of key inflammatory mediators, including *Nos2*, interleukin‐6 (*Il6*), and interleukin‐1 beta (*Il1β*), were found to be significantly up‐regulated in LPS‐treated *Acod1*‐deficient microglia, as compared to LPS‐treated control microglia (Figure 5E–G). Immunoblot analysis for NOS2, ELISA and LEGENDplex analysis for IL1β and IL6 confirmed at protein levels a significant increase in protein expression for NOS2 and IL6 (but not IL1β) in LPS‐treated *Acod1*‐deficient microglia, as compared to LPS‐treated control microglia (Figure 5H–J).

Collectively, these findings indicate that the IRG1/itaconate/NF2L2 signaling axis may exert a protective role against LPS‐induced pro‐inflammatory activation of microglia. Our results suggest that IRG1 modulates microglial inflammatory responses, positioning it as a key regulator of microglial activation and function during inflammatory conditions.

### 3.5 Repression of IRG1 Expression in Microglia Decreases LPS‐Induced Glycolytic Metabolism

**FIGURE 6:**
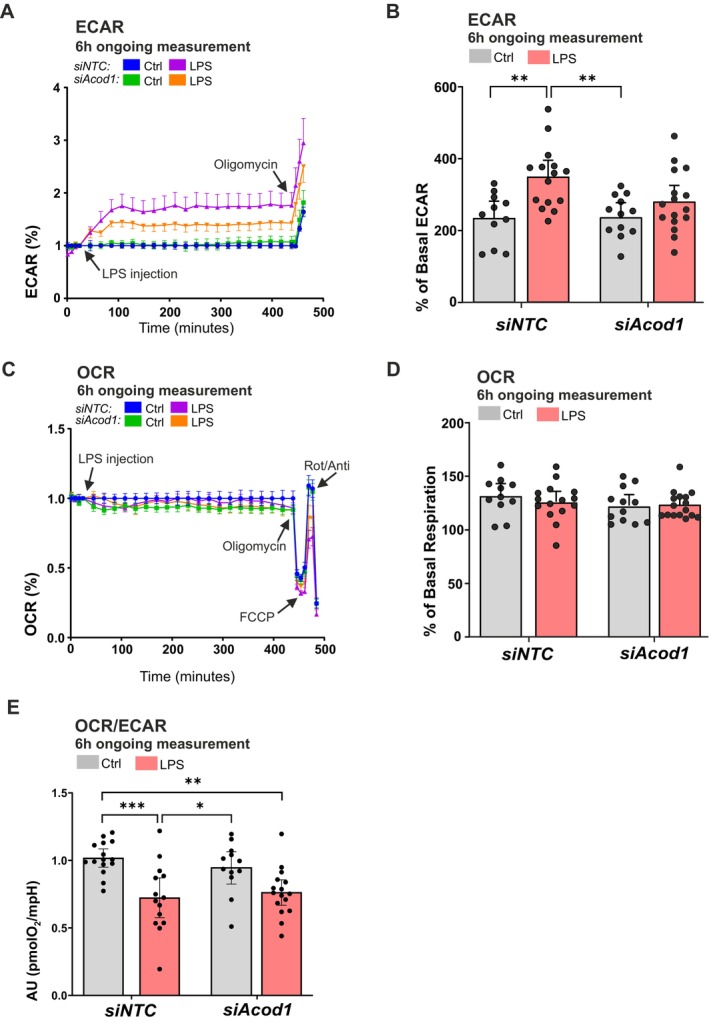
*Acod1* gene attenuation alters microglial metabolic response upon LPS stimulation. Metabolic changes were detected over 6 h of ongoing LPS treatment in the Seahorse analyzer. (A) Representative ECAR analysis curve in BV2 microglia transfected with *siAcod1* or *siNTC* and treated with LPS (100 ng/mL, 6 h) with (B) quantification of ECAR baseline. (C) analysis curve with (D) quantification to baseline, and (E) OCR/ECAR ratio for 6h of ongoing measurement in the Seahorse analyzer. Data are mean ± SEM from multiple *n =* 12 independent measurements from *N =* 2 independent biological replicates. Statistical annotations *p < 0.05; **p < 0.01; for the indicated comparison. More detailed information about sample size and used statistical tests can be found in Table S4.

To assess whether repression of *Acod1* gene expression in microglia could impact cellular metabolism, Seahorse analysis was performed with BV2 microglia transfected with either *siNTC* or *siAcod1* followed by LPS activation (Figure 6). As no significant differences in metabolic responses were detected between LPS pre‐treatment and administration shown in Figure 4, subsequent experiments in BV2 were performed with LPS added during the assay to enable real‐time assessment of the full dynamic metabolic response in microglia. Consistent with previous findings (Figure 4A,B), basal ECAR levels were significantly elevated in LPS‐stimulated *siNTC* BV2 microglia compared to unstimulated *siNTC* cells (Figure 6A,B). A significant increase of ECAR is observed in LPS‐treated *siNTC* cells after oligomycin injection, as observed before (Figures S4D and S6A). Notably, attenuation of *Acod1* gene expression rescues in part the significant up‐regulation of LPS‐treated ECAR levels, with *siAcod1* cells showing no significant difference in ECAR between LPS‐treated and untreated conditions (Figure 6B). A similar trend is observed after oligomycin treatment when *siAcod1* attenuation shows minimal changes of ECAR after LPS treatment compared to untreated *siNTC* controls (Figure S6A). As previously observed (Figure 4), basal OCR levels remain unchanged following LPS stimulation (Figure 6C,D), while oligomycin and FCCP significantly decrease OCR in LPS‐treated *siNTC* cells (Figures S5B,C,E,F, S6B,C). In contrast, OCR measurements in *siAcod1* cells showed no difference between LPS‐treated and untreated groups, and both were comparable to *siNTC* controls (Figure S6B,C).

The inability of LPS‐stimulated *siAcod1* cells to elevate ECAR to the levels observed in LPS‐stimulated *siNTC* cells (Figure 6B), combined with unchanged basal OCR (Figure 6D), resulted in the loss of a significant difference in the OCR/ECAR ratio between LPS‐treated and untreated *siAcod1* cells, which is present in the *siNTC* group (Figure 6E).

These findings underscore the crucial role of IRG1 in the regulation of microglial metabolic reprogramming during LPS‐stimulated inflammation. Suppression of IRG1 expression decreases LPS‐induced aerobic glycolysis and indicates a blunted metabolic response to LPS.

### 3.6 Repression of NF2L2/NRF2 Expression in Microglia Increases LPS‐Induced ROS Production and Neurotoxicity

**FIGURE 7:**
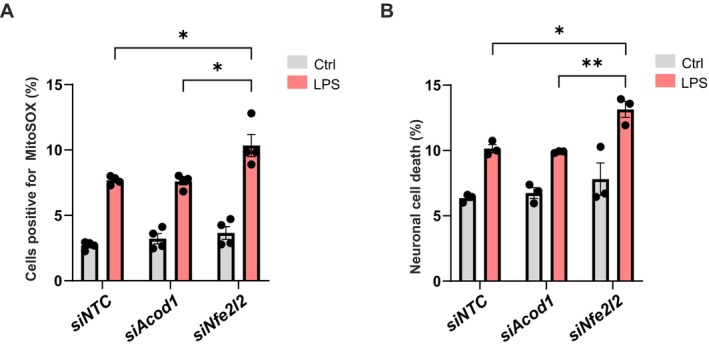
*Nef2l2*, but not *Acod1*, gene silencing leads to increased microglial ROS production and neurotoxicity after LPS stimulation. (A) Measurement of superoxide production by MitoSOX fluorescent probe using flow cytometry for BV2 microglia transfected with *siAcod1*, *siNfe2l2* or *siNTC* and treated with LPS (100 ng/mL, 6 h). (B) Neurotoxic effect of direct coculture of dopaminergic MN9D neurons in presence of BV2 microglia transfected with *siAcod1*, *siNfe2l2* or *siNTC* and treated with LPS (100 ng/mL, 24 h). CellTracker Green fluorescent labeled MN9D neurons with abnormal or damaged nuclei were counted and presented as total percentage of cell death. Data are mean ± SEM from *N =* 4 (A) and *N =* (3) different passages. Statistical annotations *p < 0.05; **p < 0.01; for the indicated comparison. More detailed information about sample size and used statistical tests can be found in Table S4.

IRG1 mediated itaconate production culminates in the activation of the NF2L2/NRF2 transcription factor, and as a consequence, the regulation of NF2L2/NRF2's target genes such as antioxidant genes and cytokines, leading to the neutralization of cellular reactive oxygen species (ROS) and reduced pro‐inflammatory response and its associated neurotoxicity (Morgenstern et al. 2024; O'Rourke et al. 2024). To get information about the importance of NF2L2/NRF2 induction during LPS‐induced microglial activation, its expression was reduced by making use of a small interfering RNAs pool targeting *Nfe2l2* gene expression. The efficiency of the siRNA‐mediated repression in *Nfe2l2* gene expression in BV2 microglia is shown in Figure 5C. Immunoblot analysis for NF2L2/NRF2 confirmed the reduction in expression at the protein level (Figure 5D). LEGENDplex analysis for IL1β and IL6 confirmed at the protein levels a significant increase in protein expression for IL1β and IL6 in LPS‐treated NF2L2/NRF2‐deficient microglia, as compared to LPS‐treated control microglia (Figure 5K,L). Considering that NF2L2/NRF2 is considered a master regulator of neutralizing cellular ROS and restoring redox balance (Morgenstern et al. 2024), reduction in its expression in microglia could lead to enhanced cellular ROS production, which in turn can lead to increased neurotoxicity when those cells are challenged with an inflammogen like LPS (Burguillos et al. 2011). Microglia were transfected with siRNA pools targeting *Acod1* or *Nfe2l2* gene expression and thereafter exposed to LPS to induce an inflammatory response. MitoSOX, a fluorescent indicator of mitochondrial superoxide, was used to measure mitochondrial ROS production. As previously reported, LPS treatment alone in control microglia resulted in an increase in mitochondrial ROS. Whereas targeting *Acod1* gene expression did not affect LPS treatment‐induced ROS production in microglia, targeting *Nfe2l2* gene expression significantly promoted the production of ROS in LPS‐treated microglia (Figure 7A).

ROS originating from LPS‐treated microglia, as well as pro‐inflammatory cytokines, e.g., IL1β and IL6 (see Figure 5F,G) can adversely affect neuronal viability (Minogue et al. 2012). To assess the impact of reduced *Acod1* and *Nfe2l2* gene expressions on the neurotoxicity of LPS‐treated microglia, direct co‐culture experiments were performed with dopaminergic MN9D neuronal cells. As previously reported, after 24 h exposure of the microglia–neurons co‐culture to LPS, increased neuronal cell death was observed (Burguillos et al. 2011). Likewise, what was observed for ROS production, whereas targeting microglial *Acod1* gene expression did not affect LPS treatment induced microglia‐mediated neurotoxicity, targeting microglial *Nfe2l2* gene expression significantly promoted the cell death of neurons in the microglia–neuron co‐culture challenged with LPS (Figure 7B).

Collectively, these findings suggest that the activation of NF2L2/NRF2 in microglia upon stimulation with the pro‐inflammatory stimulus, LPS, works as a feedback negative loop that moderates the induction of pro‐inflammatory cytokines, ROS production, and thereby the associated neurotoxicity. Concurring, counteracting NF2L2/NRF2 functions in microglia exacerbates the microglial pro‐inflammatory response and thereby neuronal dismissal.

### 3.7 *Acod1* Silencing Increases Cytokine Expression and Metabolic Balance in LPS‐Stimulated Primary Microglia

**FIGURE 8:**
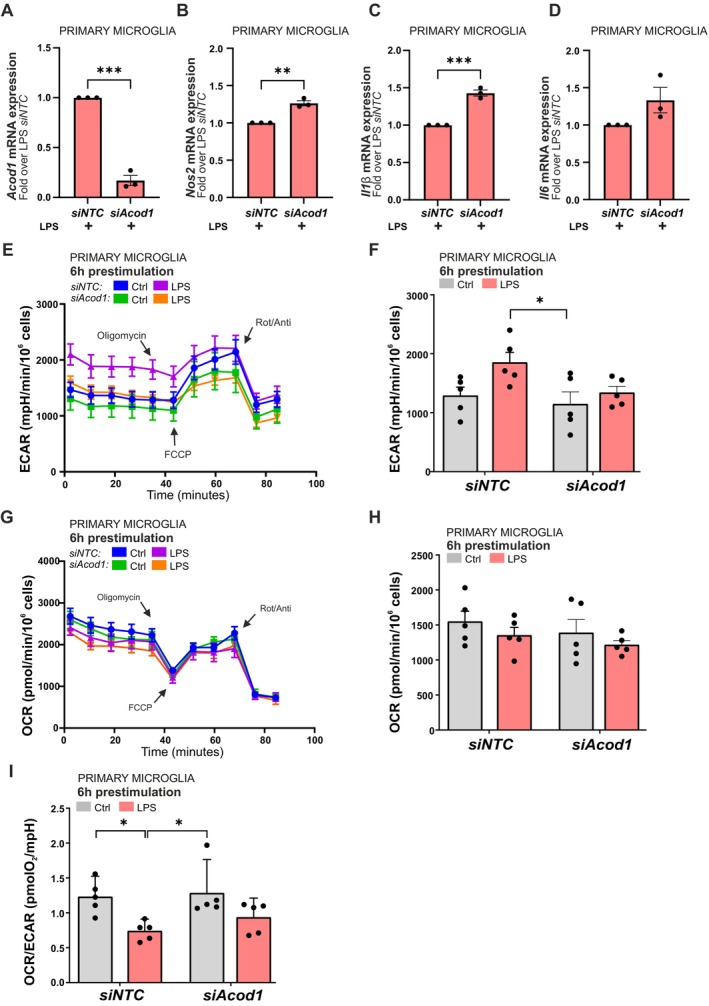
*Acod1* gene silencing enhances reactivity and alters metabolic response to LPS‐induced inflammation in murine primary microglia. (A) RT‐qPCR analysis of *Acod1* expression in LPS‐treated primary microglia transfected with *siAcod1* or *siNTC*. Gene expression of (B) *Nos2*, (C) *Il1β*, and (D) *Il6* under the same conditions. Seahorse analysis was performed in murine primary microglia after 6‐h stimulation with LPS (100 ng/mL) at normal culture condition, with subsequent Seahorse analysis showing (E) ECAR analysis curve of primary microglia transfected with *siAcod1* or *siNTC* and (F) ECAR quantification. (G) OCR analysis curve with (H) quantification of OCR, and (I) OCR/ECAR ratio. Data are mean ± SEM from *n =* 10 mouse brains each from *N =* 3 independent isolations (A–D) and from *n =* 10 mouse brains each from *N =* 1 independent isolation (E–I). Statistical annotations *p < 0.05; **p < 0.01; for the indicated comparison. More detailed information about sample size and statistical tests can be found in Table S4.

To confirm the metabolic responses observed in BV2 cells, we extended our analysis to primary murine microglia. First, we examined how *Acod1* silencing affected LPS‐induced inflammatory responses. Primary microglia were transfected with control (*siNTC*) or *Acod1*‐targeting siRNA and subsequently stimulated with LPS for 6 h. Quantitative PCR analysis revealed that *Acod1* silencing resulted in a significant reduction of *Acod1* transcript levels after LPS stimulation (Figure 8A). Notably, *Acod1*‐silenced primary microglia exhibited an increase in *Nos2*, *Il1b*, and *Il6* mRNA expression compared to *siNTC* controls upon LPS exposure (Figure 8B–D). These findings indicate that *Acod1* attenuation led to a mild but significant increase in the expression of LPS‐driven inflammatory genes in primary microglia, consistent with our observations in BV2 cells.

To investigate whether *Acod1* also modulates the metabolic response of primary microglia to LPS, we performed Seahorse analysis, similar to BV2 cells. Due to the sensitive nature of primary cells, we decided to utilize pre‐stimulation with LPS (100 ng/mL, 6 h) at normal culture conditions with subsequent analysis. LPS‐stimulated *siNTC* primary microglia exhibited an increase in ECAR, indicative of a shift toward glycolysis, like BV2 microglia. Silenced *Acod1* moderately reduced the LPS‐induced increase in ECAR, bringing levels closer to those observed in unstimulated *siAcod1* or *siNTC*‐transfected primary microglia (Figure 8E,F). The same response is observed in ECAR after oligomycin addition when *siNTC* LPS‐treated primary cells show greater up‐regulation of extracellular acidification compared to unstimulated controls as it is after *Acod1* silencing (Figure S7A). This observation agrees with trends observed for BV2 with silenced *Acod1* (Figure S6A). In primary microglia, we observe a mild, but not significant, decrease in basal OCR levels after LPS treatment in *siNTC* and *siAcod1* groups (Figure 8G,H). However, after oligomycin and FCCP treatment, OCR levels are more similar to those in BV2 microglia, as *siAcod1* treated primary microglia after LPS stimulation lose the ability to further decrease OCR levels (Figure S7B,C).

To further characterize the metabolic effects of LPS stimulation and *Acod1* silencing in primary microglia, we analyzed the OCR to ECAR ratio. LPS treatment led to a significant decrease in the OCR/ECAR ratio in the *siNTC* control group, while a blunted LPS response is seen in *siAcod1* cells (Figure 8I).

Together, these results demonstrate that *Acod1* gene attenuation in primary microglia amplifies the LPS‐induced inflammatory response and blunts glycolytic responses, mirroring the metabolic phenotype observed in BV2 cells (Figures 4 and 6). These data support a conserved role for *Acod1* in restraining pro‐inflammatory activation and modulating immunometabolic pathways in microglia.

## 4 Discussion

Metabolic profiling of microglia activated toward distinctive reactive states has the potential to provide unbiased in‐depth exploration of changes in metabolism due to metabolic reprogramming and provide unique mechanistic insights about the role of metabolites in the control of these various microglial phenotypes and their associated functions.

In this work, polar metabolic profiling revealed that LPS‐treated and IL4‐treated microglia exhibit distinct metabolite signatures. Indeed, LPS treatment, but not IL4 treatment, elicited pronounced up‐regulation of d‐Fructose bisphosphate and phosphoenolpyruvic acid, key intermediates in glycolysis. Furthermore, the interconnected glycerol phosphate shunt was also represented in LPS‐treated microglia with an up‐regulation of glycerol‐3‐phosphate. A striking selective up‐regulation of endogenous itaconate, with the concomitant significant decrease in succinate, a metabolite downstream to itaconate within the TCA cycle, indicated impairment of this pathway in LPS‐stimulated microglia. In fact, high production of itaconate limits the enzymatic activity of succinate dehydrogenase (SDH), thereby affecting the flux of the TCA cycle, which we observed in the decrease in maximal respiration (O'Neill and Artyomov 2019). The metabolite profile observed in LPS‐treated microglia, absent in their IL4‐treated counterparts, implies an up‐regulation of aerobic glycolysis as an energy‐yielding pathway in microglia in response to an inflammogen. In agreement, the cellular bioenergetic analysis showed an increase in extracellular acidification rate (ECAR) in pro‐inflammatory activated microglia. Corroborating the above findings, increased microglial glycolysis in response to pro‐inflammatory stimuli, for example, LPS, lysophosphatidic acid, interferon‐γ or amyloid‐β, has been reported in various microglial cell lines, primary mouse microglia, as well as in human iPSc‐derived microglia (Sabogal‐Guáqueta et al. 2023; Voloboueva et al. 2013; Bernhart et al. 2010; Rubio‐Araiz et al. 2018; Ma et al. 2022). In vivo, in the mouse models for stroke, Alzheimer's disease, Parkinson's disease, or spinal cord injury, microglia also presented an increased glycolysis, and itaconate was even found to be up‐regulated in the murine stroke model (Loppi et al. 2023; Lu et al. 2022; McIntosh et al. 2019).

It is well‐established that numerous peripheral immune cells, including macrophages, up‐regulate glycolysis when activated. It has been proposed that glycolysis could provide these myeloid cells with a faster rate of ATP production and the generation of intermediates for cell growth and cytokine production, such as we found in the PPP pathway (Lauro and Limatola 2020). Despite originating from distinct embryonic lineages, holding mostly different locations (central versus peripheral), and exerting unique functions that distinguish them, microglia and macrophages share some similarities. Hence, one can speculate that the metabolic reprogramming in response to inflammatory challenges could exert a similar effect on these two myeloid cell populations. Supporting this idea, the LPS‐induced metabolic reprogramming toward glycolysis has been shown to promote cytokine and chemokine productions (Nair et al. 2019). LPS‐induced microglia activation state is also characterized by increased oxidative stress‐mediated neurotoxicity that relies on microglial ROS production (Burguillos et al. 2011). Glycolysis and mitochondrial ROS production are two entangled processes that regulate each other (Liemburg‐Apers et al. 2015). A high glycolytic flux has been associated with increased ROS levels in various cell types (Yu et al. 2006). In fact, we observed both increased ROS production and neurotoxicity in the glycolytic microglia in response to LPS exposure. Hence, one can speculate that the observed increase in glycolysis in microglia in response to exposure to an inflammogen contributes to various features of the pro‐inflammatory microglial reactive states, ranging from cytokine and ROS production to neurotoxicity.

Our investigations also highlight endogenous itaconate, as a metabolite whose increase upon LPS treatment defines pro‐inflammatory microglia. Itaconate is produced by IRG1, whose encoding gene, *Acod1*, was first discovered as an LPS‐induced gene in mouse macrophages (Michelucci et al. 2013; Lee et al. 1995). Itaconate mediates transcriptional effects via the alkylation of KEAP1, an NF2L2/NRF2 inhibitor that mediates its ubiquitin‐dependent proteasomal degradation, which in turn leads to the stabilization and nuclear accumulation of this transcription factor (Ahmed et al. 2017; Itoh et al. 2010). Induction of *Acod1*/IRG1 expression and stabilization of NF2L2/NRF2 were observed in our model system, hence uncovering an IRG1/itaconate/NF2L2 signaling axis regulating the complex response of microglia to an LPS challenge that includes a change in TCA cycle metabolites and a glycolytic up‐regulation, the production of mitochondrial ROS, the induction of expression of pro‐inflammatory cytokines as well as genes with antioxidant properties. Some of the effects observed in LPS‐treated microglia may appear contradictory, e.g., ROS production and antioxidant gene expression, and therefore suggest that the uncovered IRG1/itaconate/NF2L2 signaling axis could contribute to the fine‐tuned regulation of the LPS‐induced microglia pro‐inflammatory response. Our experimentation that aimed at lowering the expression of IRG1 and NF2L2/NRF2 supports the existence of an itaconate‐dependent regulatory loop. Indeed, targeting *Acod1* gene expression led to reduced glycolysis but increased expression of pro‐inflammatory associated genes, that is, *Nos2*, *Il1β* and *Il6*, in LPS‐treated microglia. However, decreased IRG1 expression did not affect ROS production or neurotoxicity associated with LPS‐treated microglia. Targeting *Nef2l2* gene expression, likewise, targeting *Acod1* gene expression, led to increased *Il1β* and *Il6* gene expression but did not influence LPS‐induced microglial ROS production and the dismissal of neurons. Collectively, these data suggest that IRG1‐produced itaconate contributes to the shift to glycolysis, ROS production, and the activation of a pro‐inflammatory transcriptional program that ultimately promotes neuronal cell death. In addition, itaconate, as an NF2L2/NRF2 positive regulator, promotes the activation of a transcriptional feedback negative loop that reduces ROS production and neurotoxicity of LPS‐stimulated microglia. Thus, itaconate appears as a fine‐tuned regulator of the microglial response to pro‐inflammatory stimuli, exerting positive and negative effects.

Itaconate, or itaconate signaling pathways, has been considered potential therapeutic targets for diseases with an inflammation component (Shi et al. 2022; Lin et al. 2021). Itaconate derivatives, dimethyl itaconate, 4‐octyl itaconate, and 4‐monoethyl itaconate, more suitable to cross the blood–brain barrier, have been proposed to be potential therapeutic agents that would exert anti‐inflammatory, immunomodulatory, and antioxidant effects and help to combat diseases or disorders with a neuroinflammatory component (Lin et al. 2021). Treating mice with 4‐octyl itaconate attenuated macrophage‐mediated inflammation induced by LPS or high cholesterol (Song et al. 2023; Xu et al. 2023; Ren et al. 2022). Treatment with dimethyl itaconate reduces in vitro LPS‐induced inflammatory response in both macrophages and microglia (Ren et al. 2022). Alternative approaches reside in the use of KEAP1–NF2L2/NRF2 protein–protein interaction inhibitors which promote NF2L2/NRF2 activation (Zou et al. 2024; Colarusso et al. 2020; Crisman et al. 2023). KEAP1–NF2L2/NRF2 inhibitors have been shown to exert antioxidant and anti‐inflammatory effects in LPS‐stimulated macrophages in vitro and in vivo (Lu et al. 2020). However, whether those IRG1/itaconate/NF2L2 axis‐related small molecules could be used to target microglia in the context of neuroinflammation remains to be established. Also considering that itaconate‐dependent signaling pathways exert both positive and negative effects on microglial inflammatory capabilities and associated neurotoxicity, one may want to develop strategies that would target itaconate functions as SDH inhibitor, NF2L2/NRF2 activator, beside other of its reported functions, for example, regulator of ATF3 (Cyclic AMP‐dependent transcription factor ATF‐3) and NLRP3 (NACHT, LRR and PYD domains‐containing protein 3). Our findings extend and complement earlier studies of microglial itaconate biology. (Voloboueva et al. 2013; Chausse et al. 2020; Kong et al. 2024; Liu et al. 2025) While prior work demonstrated that itaconate can exert anti‐inflammatory or neuroprotective effects, these studies focused on ex vivo slice cultures, systemic administration of itaconate, or injury models over extended timeframes. In contrast, our study directly measures endogenous itaconate production in vitro during the early activation phase, compares pro‐ and anti‐inflammatory conditions side by side, and links this metabolic response to Nrf2 stabilization. This approach reveals a mechanistic IRG1/itaconate/Nrf2 feedback loop that was not previously characterized, thereby providing new insights into how microglia regulate oxidative stress and neurotoxicity during inflammatory activation.

In this study, we used BV2 cells as a model to investigate microglial activation and metabolic reprogramming. BV2 cells are a one‐week‐old immortalized murine microglial cell that retains many functional and phenotypic characteristics of primary microglia, including their ability to respond to pro‐ and anti‐inflammatory stimuli, secrete cytokines, produce reactive oxygen species, and perform phagocytosis comparable to primary microglia or acutely isolated microglia (Henn et al. 2009; Luan et al. 2022). Their ease of culture, high proliferative capacity, and suitability for genetic manipulation, such as siRNA transfection, make them a widely used and practical model for in vitro studies of microglial biology. However, it is important to acknowledge the limitations of using BV2 cells as an experimental model. Despite high similarity with primary microglia, they may exhibit altered signaling pathways and metabolic profiles compared to primary microglia, and they do not fully recapitulate the complexity of microglial responses in vivo, especially the effect of other neuronal cells. Although these limitations, BV2 cells provide a valuable step for investigating molecular mechanisms under highly controlled experimental conditions that cannot be reached in vivo. To address these limitations and strengthen the physiological relevance of our findings, we additionally performed key experiments in primary murine microglia, which confirmed the observations made in BV2 cells.

In conclusion, our metabolic profiling, together with bioenergetic as well as functional cellular analyses, reveals that microglia activated toward an inflammatory phenotype, in response to IRG1‐produced itaconate, increase aerobic glycolysis and the production of pro‐inflammatory cytokines and ROS, but that one of the outcomes of this microglial activation, that is, neurotoxicity, is reduced via the parallel induction of the itaconate‐dependent activation of the NF2L2/NRF2 transcriptional program that lowers the oxidative stress in those cells.

## Author Contributions

**Pinelopi Engskog‐Vlachos:** conceptualization, methodology, data curation, investigation, formal analysis, visualization, writing – original draft. **Mikael K. R. Engskog:** conceptualization, data curation, formal analysis, visualization, writing – original draft, methodology, investigation. **Martin Skandik:** data curation, formal analysis, visualization, writing – original draft, investigation. **Kathleen Grabert:** formal analysis, writing – review and editing, data curation, investigation. **Noah Moruzzi:** data curation, formal analysis, methodology, visualization, writing – review and editing, investigation. **Marie‐Kim St‐Pierre:** investigation, methodology, writing – review and editing. **Ahmed M. Osman:** investigation, writing – review and editing. **Theodora Sylaidi:** investigation, methodology, writing – review and editing. **Klas Blomgren:** writing – review and editing, project administration, supervision. **Per‐Olof Berggren:** supervision, project administration, writing – review and editing. **Bertrand Joseph:** writing – original draft, conceptualization, supervision, funding acquisition, project administration.

## Conflicts of Interest

P.‐O.B. is co‐founder and CEO of Biocrine AB. The other authors declare no competing interests.

## Peer Review

The peer review history for this article is available at https://www.webofscience.com/api/gateway/wos/peer‐review/10.1111/jnc.70219.

## Supporting information


**Figure S1:** jnc70219‐sup‐0001‐Supinfo.pdf.

## Data Availability

The data that support the findings of this study are available on request from the corresponding author.
