# Supplementary material for: Metabolic Profiling Reveals a Glycolytic Shift and an IRG1/Itaconate/NF2L2 Axis Regulating Neurotoxic Oxidative Stress in Inflammatory Microglia
Source: J Neurochem. 2025 Sep 5;169(9):e70219. doi: 10.1111/jnc.70219 (PMC12413559; doi:10.1111/jnc.70219)
Supplement: Supplementary file 1 — Figure S1: jnc70219‐sup‐0001‐Supinfo.pdf. [file JNC-169-0-s001.pdf]

Metabolic profiling reveals a glycolytic shift and an IRG1/itaconate/NF2L2 axis regulating neurotoxic oxidative stress in inflammatory microglia.

Pinelopi Engskog-Vlachos[1,6] Mikael K.R. Engskog[2,6], Martin Skandik[1], Kathleen Grabert[1], Noah Moruzzi[4], Marie-Kim St-Pierre[1], Ahmed M Osman[5], Theodora Sylaidi[5], Klas Blomgren[5], Per-Olof Berggren[4] and Bertrand Joseph[1,3,7,*]

[1] Institute of Environmental Medicine, Toxicology Unit, Karolinska Institutet, Stockholm, Sweden.

[2] Department of Medicinal Chemistry, Uppsala University, Uppsala, Sweden.

[3] Center for Neuromusculoskeletal Restorative Medicine, Shui On Centre, Wan Chai, Hong Kong.

[4] The Rolf Luft Research Center for Diabetes and Endocrinology, Karolinska Institutet, Stockholm, Sweden

[5] Department of Women's and Children's Health, Karolinska Institutet, Stockholm, Sweden.

[6] These authors contributed equally.

[7] Lead contact.

[*] Correspondance: bertrand.joseph@ki.se.

Supplementary Figure 1

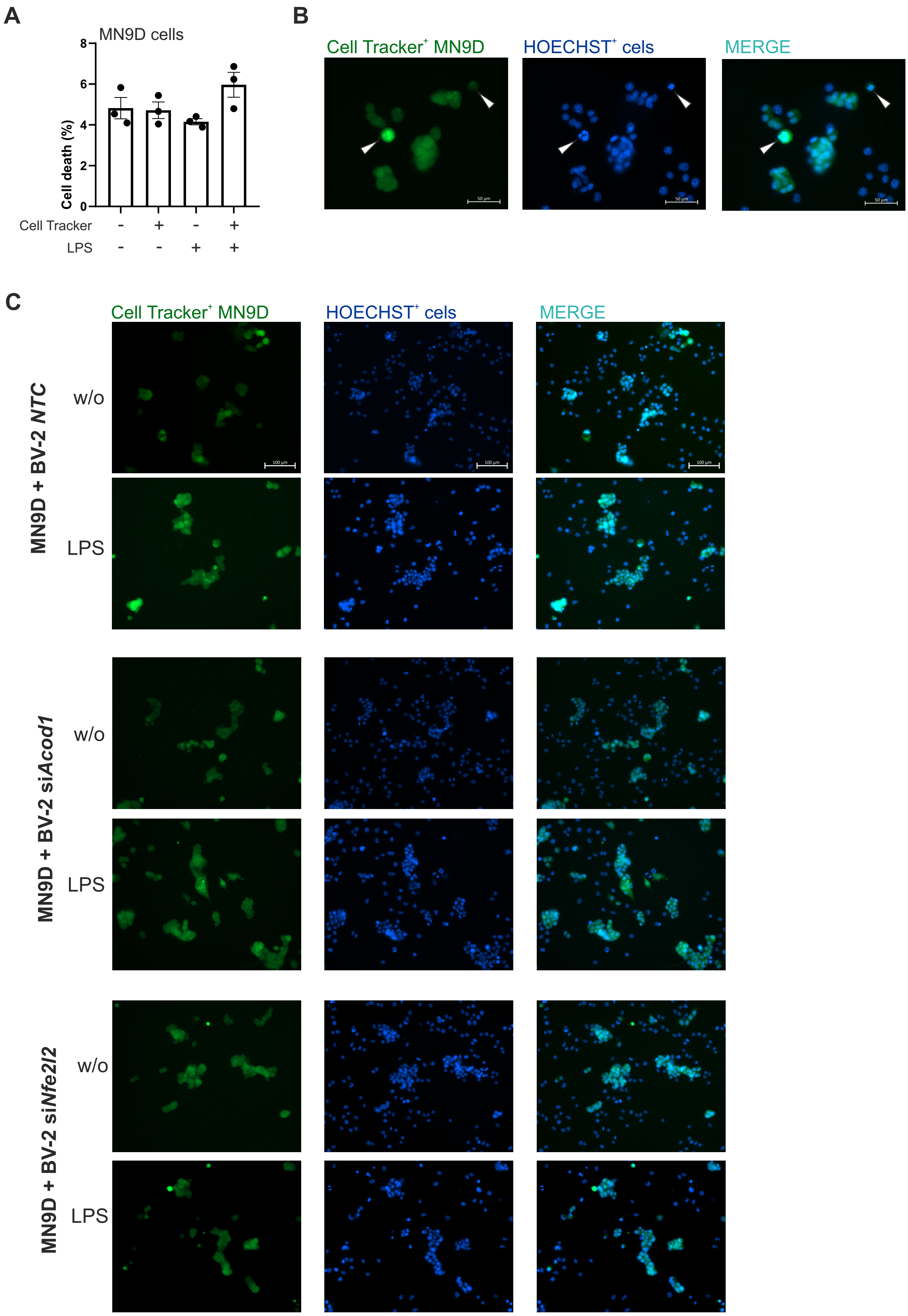

Supplementary Figure 1. (**A**) MN9D neuronal cells exposed to CellTracker, LPS, and their combination to evaluate non-toxic effect of experimental set up; (**B**) Representative microscopic images highlighting damaged MN9D neural nuclei that was counted; (**C**) Representative microscopic images of MN9D and BV-2 microglia for all experimental conditions.

Supplementary Figure 2

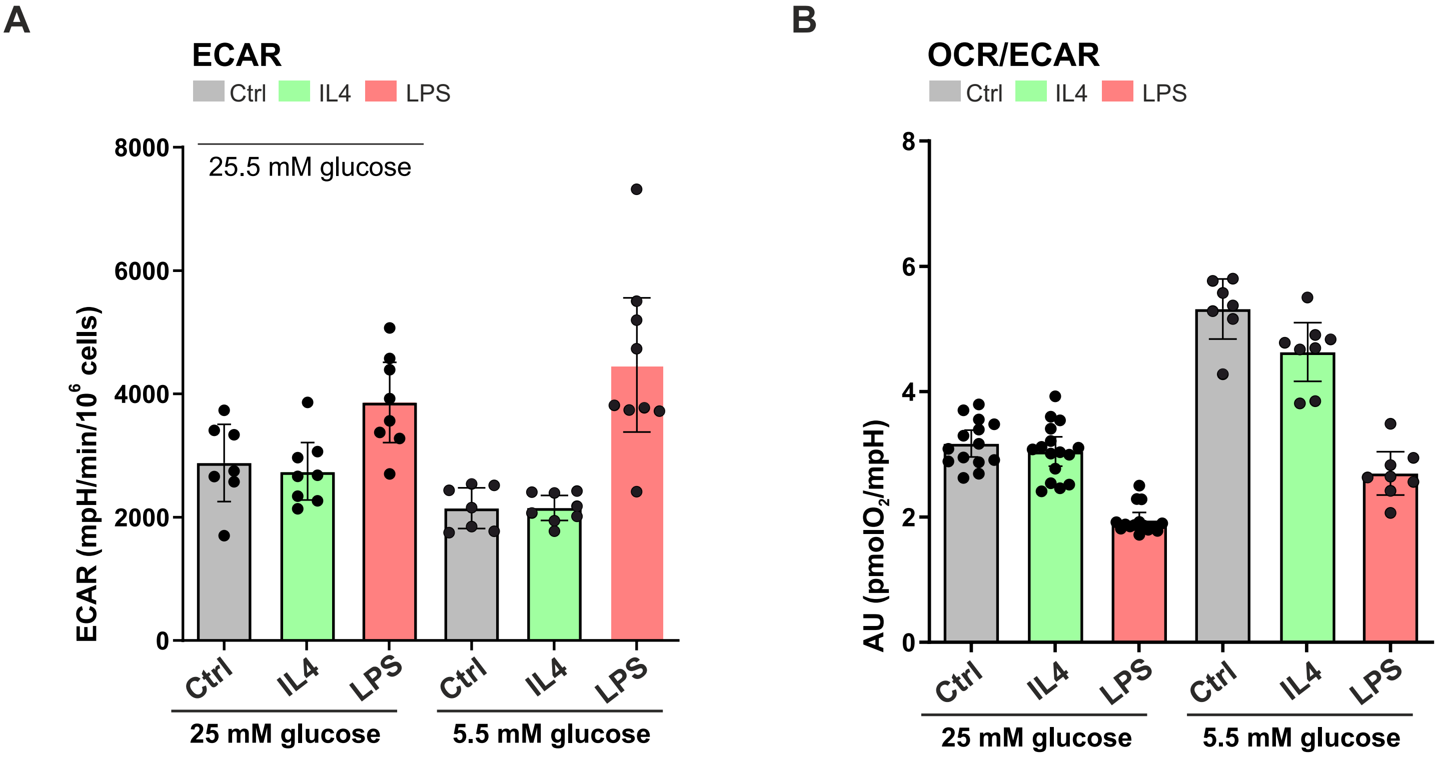

Supplementary Figure 2. Analysis of glucose concnetration effects on Bv2 microglial metabolism. (A) Extracellular acidification rate (ECAR) in BV2 microglia cultured under high (25 mM) or physiological (5.5 mM) glucose conditions at baseline and following LPS or IL-4 stimulation.(B) OCR/ECAR ratio illustrating a shift toward increased respiratory activity under lower glucose conditions. In both glucose conditions, LPS stimulation induces metabolic changes that are independent of glucose availability.

Supplementary Figure 3

Table depicting filtering from detection to metabolites

	POSTIVE MODE		NEGATIVE MODE	
Stable features[a]	3060 out of 4614 features		2850 out of 4489 features	
Treatment	IL4	LPS	IL4	LPS
Selected features from Volcano plot [b]	1189	1079	582	540
Up-regulated *vs* Ctrl	810	618	418	430
Down-regulated *vs* Ctrl	379	461	164	110
**Significant metabolites[c]**	**37**	**39**	**52**	**46**

a) Retention time > 45 s, CV < 20%
b) FC threshold 1.5
c) Annotated metabolites significant according to the post hoc tukey test, mzdi ff> 0.02 Da

Supplementary Figure 3. Table summarizes the number of features identified, filtered, and deemed analytically stable based on retention time and CV criteria, as well as the number of features meeting significance thresholds for differential expression across groups.

Supplementary Figure 4

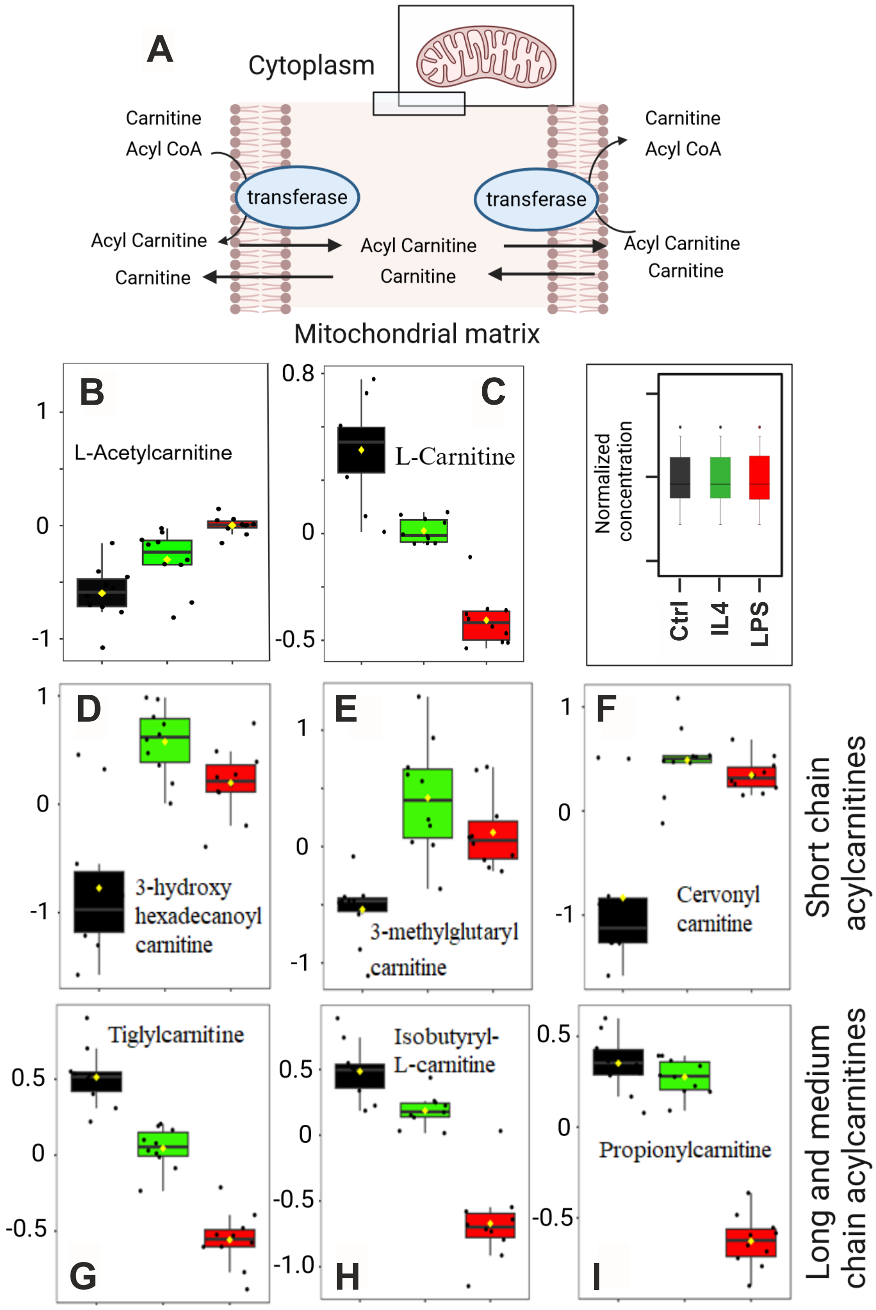

Supplementary Figure 4. (**A**) Overview scheme of significant metabolic alterations in carnitin-mediated transport through mitochondrial membrane; (**B**) L-Acetylcarnitine; (**C**) L-Carnitine; (**D**) 3-hydroxy hexadecanoyl carnitine; (**E**) 3-methylglutaryl carnitine; (**F**) Cervonyl carnitine; (**G**) Tiglylcarnitine; (**H**) Isobutyryl-L-carnitine; (**I**) propionylcarnitine. Graphs represent relative concentrations of metabolites.

Supplementary Figure 5

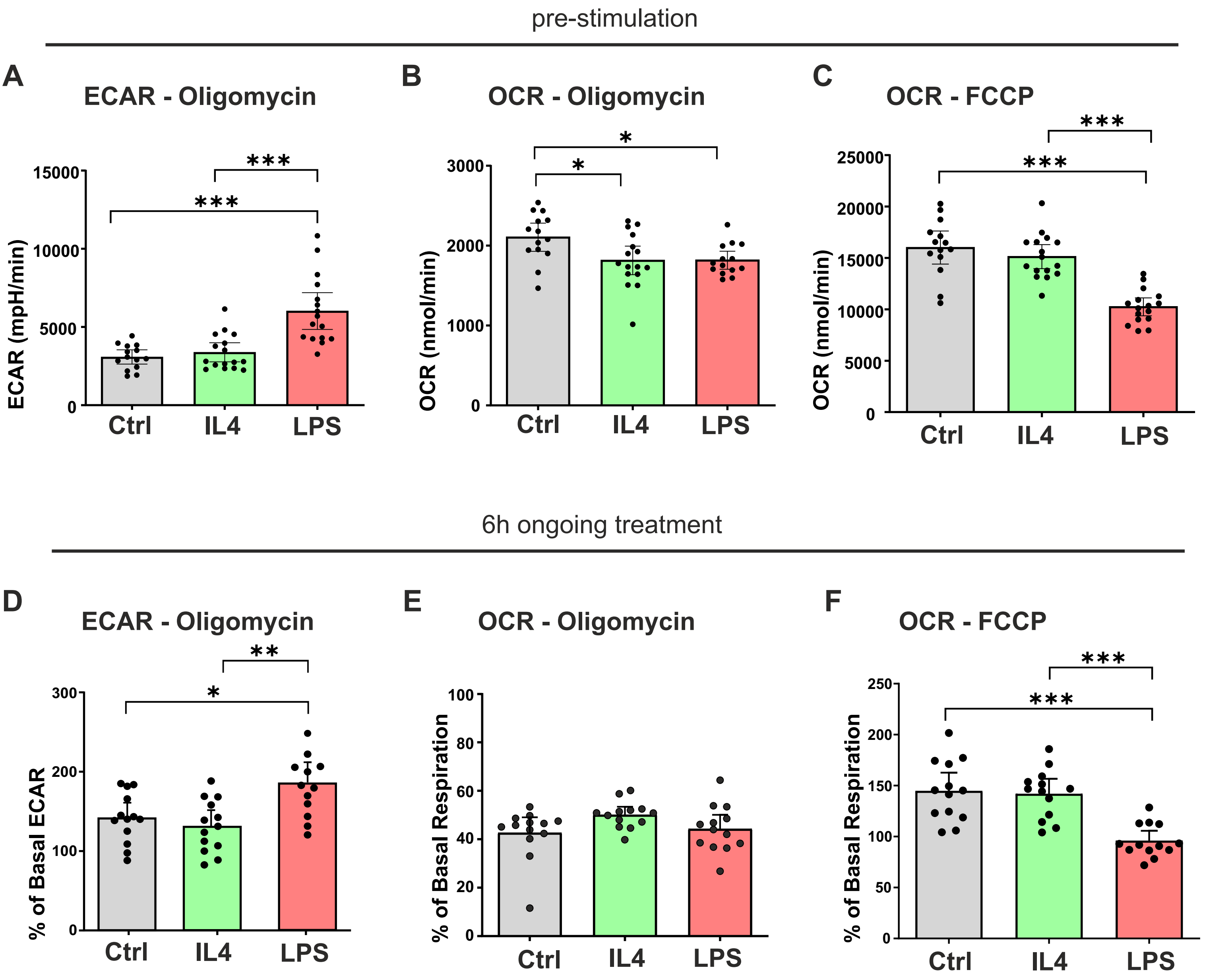

Supplementary Figure 5. Seahorse analysis of ECAR and OCR in BV2 microglia untreated, after 6h pre-stimulation with LPS (100ng/ml), and IL4 (20ng/ml) showing (**A**) ECAR after oligomycin addition, (**B**) OCR after oligomycin addition, (**C**) OCR after FCCP treatment. Seahorse analysis performed as ongoing mesurement covering whole stimulation time (6h) with LPS and IL4 showing (**D**) ECAR after oligomycin addition, (**E**) OCR after oligomycin addition, (**F**) OCR after FCCP treatment.

Supplementary Figure 6

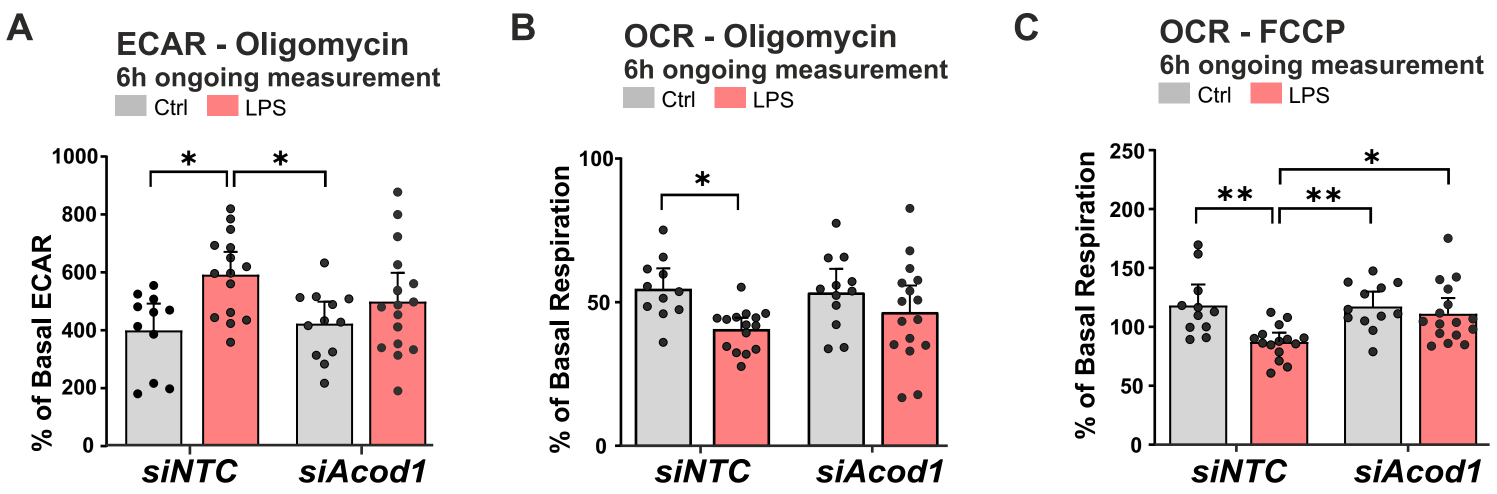

Supplementary Figure 6. Seahorse analysis of ECAR and OCR in BV2 microglia untreated, treated with LPS (100ng/ml), and IL4 (20ng/ml) during ongoing 6-hour long measurement in Seahorse analyzer showing (**A**) ECAR after oligomycin addition, (**B**) OCR after oligomycin addition, (**C**) OCR after FCCP treatment.

Supplementary Figure 7

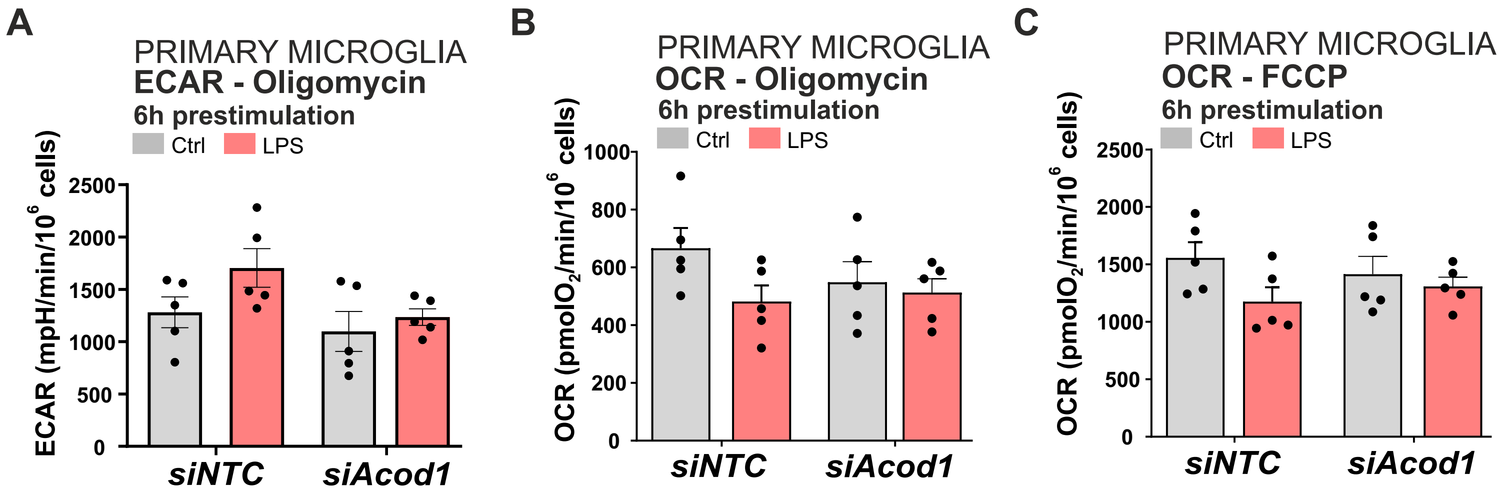

Supplementary Figure 7. Seahorse analysis of ECAR and OCR in primary microglia untreated, after 6h pre-stimulation with LPS (100ng/ml), and IL4 (20ng/ml) showing (**A**) ECAR after oligomycin addition, (**B**) OCR after oligomycin addition, (**C**) OCR after FCCP treatment.

Uncropped Immunoblots

Figure 1A

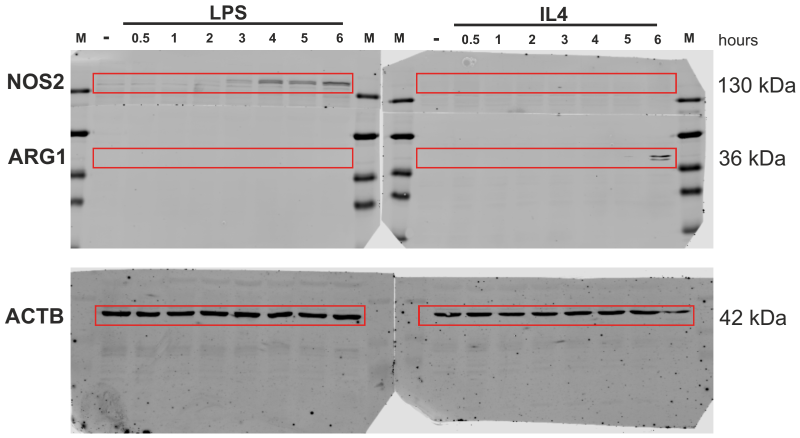

Figure 5A

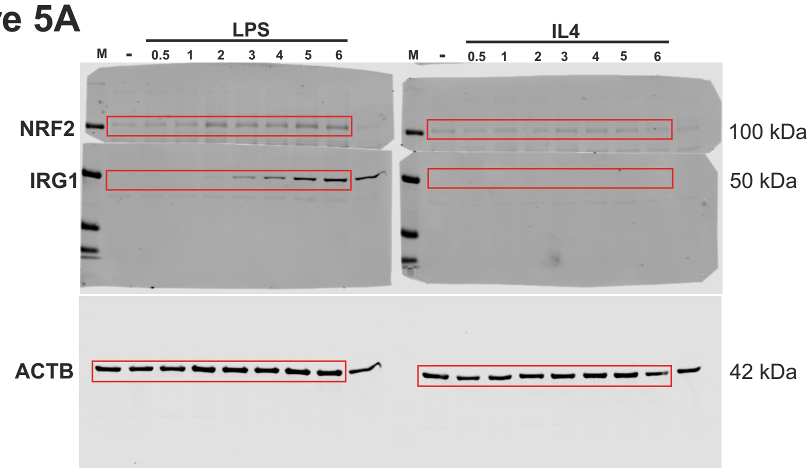

Figure 5D

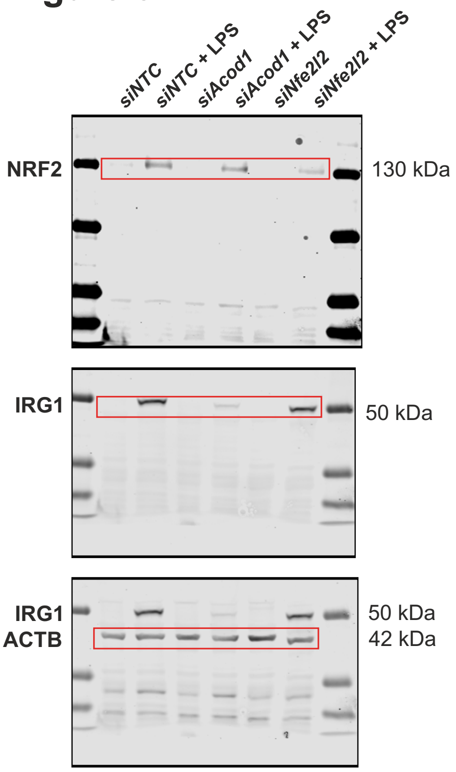

Figure 5H

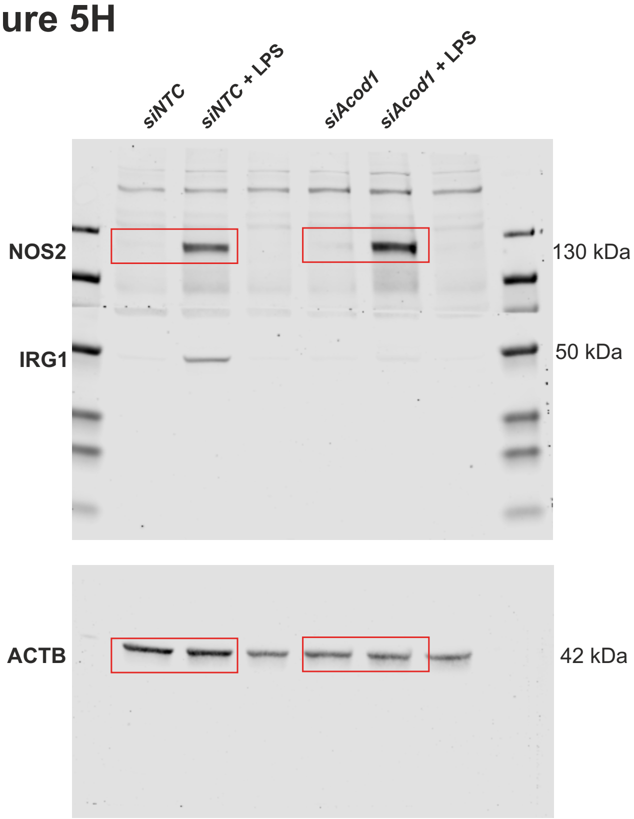

Supplementary Table 1 | Antibodies used in this study

REAGENT or RESOURCE	SOURCE	IDENTIFIER
Antibodies		
Mouse monoclonal anti-Actin (Clone AC-40)	Sigma-Aldrich	Cat# A3853<br>RRID: AB_2621137
Rabbit monoclonal anti-NRF2 (E5F1A)	Cell Signaling Technology	Cat# 20733<br>RRID: AB_2934224
Rabbit monoclonal anti-IRG1	Abcam	Cat# 222411<br>RRID:AB_2868510
Rabbit monoclonal anti-NOS2	Cell Signaling Technology	Cat# 13120,<br>RRID:AB_2687529
Goat polyclonal anti-ARG1 (V-20)	Santa Cruz Biotechnology	Cat# sc-18354,<br>RRID:AB_2227469
IRDye 800CW Goat anti-Mouse IgG	LI-COR Biosciences	Cat# 926-32210,<br>RRID:AB_621842
IRDye 680RD Goat anti-Rabbit IgG	LI-COR Biosciences	Cat# 926-68071,<br>RRID:AB_10956166
IRDye 800CW Donkey anti-Goat IgG	LI-COR Biosciences	Cat# 926-32214,<br>RRID:AB_621846)

Supplementary Table 2 | ON-TARGETplus SMART pool small interfering RNAs used in this study

ON-TARGET plus SMARTpools siRNAs	Company
**Oligonucleotides**<br>siRNA targeting sequence: ON -TARGET plus SMARTpools siRNAs: Acod1, mouse, NM_008392<br>Seq:<br>GCACAGAAGUGUUCCAUAA<br>GAAAUAAGCAUCACUCUAA<br>GAGCUUUGCUGGUAUGAUU<br>GAAAGUGAACCACCUGACA<br><br>siRNA targeting sequence: ON -TARGET plus SMARTpools siRNAs: Nfe2l2, mouse, NM_010902<br>Seq:<br>ACUCAAAUCCCACCUUAAA<br>UGGAGUAAGUCGAGAAGUG<br>CAUGUUACGUGAUGAGGAU<br>GGACAGCAAUUACCAUUUU<br><br>siRNA targeting sequence: ON -TARGET plus SMARTpools siRNAs: Non-targeting (NTC) siRNA pool<br>Seq:<br>UGGUUUACAUGUCGACUAA<br>UGGUUUACAUGUUGUGUGA<br>UGGUUUACAUGUUUUCUGA<br>UGGUUUACAUGUUUUCCUA	Dharmacon

Supplementary Table 3 | Primer sequences

All sequences are given 5´to 3´

**KiCqStart Primers, Sigma-Aldrich**	**Forward primer**	**Reverse primer**
Primer: *Actb* (mouse)	Forward: GATGTATGAAGGCTTTGGTC	Reverse: TGTGCACTTTTATTGGTCTC
Primer: *Acod1* (mouse)	Forward: TGTTAATGGTGTTGCTGTTC	Reverse: CCTGTACTTCAATACCAACG
Primer: *Nef2l2* (mouse)	Forward: CAGAGACATTCCCATTTGTAG	Reverse: ATTCGGGAATGGAAAATAGC
Primer: *Il1β* (mouse)	Forward: GCTGCTTCCAAACCTTTGAC	Reverse: TTCTCCACAGCCACAATGAG
Primer: *Il6* (mouse)	Forward: GGACCAAGACCATCCAATTC	Reverse: GGCATAACGCACTAGGTTTG
Primer: *Nos2* (mouse)	Forward: GCGGAGTGACGGCAAACATGAC	Reverse: AGGTCGATGCACAACTGGGTGAAC
